# Transcriptomic, proteomic and metabolic changes in *Arabidopsis thaliana* leaves after the onset of illumination

**DOI:** 10.1186/s12870-016-0726-3

**Published:** 2016-02-11

**Authors:** Chao Liang, Shifeng Cheng, Youjun Zhang, Yuzhe Sun, Alisdair R. Fernie, Kang Kang, Gianni Panagiotou, Clive Lo, Boon Leong Lim

**Affiliations:** School of Biological Sciences, The University of Hong Kong, Pokfulam, Hong Kong, China; Max Planck Institute of Molecular Plant Physiology, Am Mühlenberg 1, 14476 Potsdam-Golm, Germany; State Key Laboratory of Agrobiotechnology, The Chinese University of Hong Kong, Shatin, Hong Kong, China

**Keywords:** ATP, Chloroplast, Mitochondria, Metabolomics, Proteomics, Transcriptomics

## Abstract

**Background:**

Light plays an important role in plant growth and development. In this study, the impact of light on physiology of 20-d-old Arabidopsis leaves was examined through transcriptomic, proteomic and metabolomic analysis. Since the energy-generating electron transport chains in chloroplasts and mitochondria are encoded by both nuclear and organellar genomes, sequencing total RNA after removal of ribosomal RNAs provides essential information on transcription of organellar genomes. The changes in the levels of ADP, ATP, NADP^+^, NADPH and 41 metabolites upon illumination were also quantified.

**Results:**

Upon illumination, while the transcription of the genes encoded by the plastid genome did not change significantly, the transcription of nuclear genes encoding different functional complexes in the photosystem are differentially regulated whereas members of the same complex are co-regulated with each other. The abundance of mRNAs and proteins encoded by all three genomes are, however, not always positively correlated. One such example is the negative correlation between mRNA and protein abundances of the photosystem components, which reflects the importance of post-transcriptional regulation in plant physiology.

**Conclusion:**

This study provides systems-wide datasets which allow plant researchers to examine the changes in leaf transcriptomes, proteomes and key metabolites upon illumination and to determine whether there are any correlations between changes in transcript and protein abundances of a particular gene or pathway upon illumination. The integration of data of the organelles and the photosystems, Calvin-Benson cycle, carbohydrate metabolism, glycolysis, the tricarboxylic acid cycle and respiratory chain, thereby provides a more complete picture to the changes in plant physiology upon illumination than has been attained to date.

**Electronic supplementary material:**

The online version of this article (doi:10.1186/s12870-016-0726-3) contains supplementary material, which is available to authorized users.

## Background

Light is the ultimate source of energy for plant growth. During the light reaction of photosynthesis, light energy is used to drive the electron flow from water to NAPD^+^, and during this process, a proton gradient is established across the thylakoid membrane for ATP formation. Photosynthesis thus provides energy (ATP) and reducing power to plants, which exert great impacts on plant physiology. Information on the effects of light on the leaf transcriptome of Arabidopsis has been reported in previous studies. However these studies either employed homemade microarray with less than 10,000 probes [1, 2] or Affymetrix ATH1 [3] or Aligent Oligo microarrays [4]. The Affymetrix ATH1 microarray only contains 24,000 genes and the probe does not represent all the genes in the Arabidopsis nuclear genome (>30,000 genes) and no transcripts from the chloroplast and mitochondrial genomes were detected in the Aligent microarray [4, 5]. In plants, many biological processes are correlated with photosynthesis. Since chloroplasts and mitochondria are the two key power houses of plant cells and many components of the energy generating systems (photosystems in chloroplasts and respiratory chain in mitochondria) are encoded by both nuclear and organelle genomes, transcription data of organelle genomes are required to depict a clear picture on plant energy biology. In this report, whole genome transcriptomic data, including transcripts transcribed from the chloroplast and mitochondrial genomes, was obtained by RNA sequencing. Given that changes in transcript abundances are not always coherence with changes in protein levels [6, 7], the changes in leaf proteomes were also examined [8]. In addition, the changes in key leaf metabolites of *Arabidopsis thaliana,* including ATP, ADP, NADP^+^, NADPH, after the onset of illumination were also investigated. Metabolomics is now becoming an essential component of such post-genomic studies. As the measurements of changes in mRNA and protein levels cannot always directly reflect the changes in plant physiology, metabolomics provide a clear picture on plant’s energy and nutritional status [9]. The integration of these omics data is expected to give us a better understanding on the impacts of light on the physiology of plant leaves [10].

## Results

### RNA-seq and differential analyses

For each sample, nearly 65 M reads of 90 bases and 6 Gbp length sequences were obtained from deep sequencing. Total sequenced reads were mapped to both Arabidopsis TAIR 10.0 genes and genome respectively (Tables 1 and 2). Reads were sorted into two subgroups: single designated reads that mapped only once to the gene/genome location; and multiple reads mapped many times to more than one location in the gene/the genome. Approximately 75 % reads could be mapped to Arabidopsis genes. Around 60 % of reads were aligned to only one position whilst 15 % of reads were mapped to two or more positions (Table 1). However, when the reads were mapped to the Arabidopsis genome, approximately 85 % were aligned in each library. 80 % were mapped to only one position and 4 % were mapped to more than one position in the genome (Table 2). In total 29,480 expressed transcripts were detected in the RNA-sequencing data, which included 29,278 transcripts encoded by the nuclear genome, 126 transcripts encoded by the mitochondrial genome and 96 transcripts encoded by the chloroplast genome (Additional files 1, 2 and 3). The genes encoded by sequenced RNAs were classified by their functional classes and compared with those annotated in TAIR 10.0 (http://www.arabidopsis.org/portals/genAnnotation/genome_snapshot.jsp) (Table 3). Table 3 shows that in total transcripts of 23,840 nuclear genes were detected, of which 22,076 genes were sorted to protein coding class. The numbers are fewer than the number of transcripts detected (>29,000) because some genes expressed more than one splice variants. Only 3 pre-tRNAs were found in our samples most likely because most tRNAs are shorter than 90 bp.

**Table 1:** Total number of sequencing reads mapped to genes in TAIR 10.0

	T0		T1		T8	
Map to gene	Reads number	Percentage	Reads number	Percentage	Reads number	Percentage
Total Reads	65856848	100.00 %	68717410	100.00 %	66618050	100.00 %
Total Base Pairs	5927116320	100.00 %	6184566900	100.00 %	5995624500	100.00 %
Total Mapped Reads	50220939	76.26 %	54644882	79.52 %	52800526	79.26 %
Perfect match	39421448	59.86 %	43404340	63.16 %	42190196	63.33 %
<= 5 bp mismatch	10799491	16.40 %	11240542	16.36 %	10610330	15.93 %
Unique match	40502486	61.50 %	43070199	62.68 %	42066874	63.15 %
Multi-position match	9718453	14.76 %	11574683	16.84 %	10733652	16.11 %
Total Unmapped Reads	15635909	23.74 %	14072528	20.48 %	13817524	20.74 %

**Table 2:** Total number of sequencing reads mapped to genome in TAIR 10.0

	T0		T1		T8	
Map to genome	Reads number	Percentage	Reads number	Percentage	Reads number	Percentage
Total Reads	65856848	100.00 %	68717410	100.00 %	66618050	100.00 %
Total Base Pairs	5927116320	100.00 %	6184566900	100.00 %	5995624500	100.00 %
Total Mapped Reads	56308614	85.50 %	59953853	87.25 %	58351588	87.59 %
Perfect match	43870842	66.62 %	47185072	68.67 %	46153587	69.28 %
<= 5 bp mismatch	12437772	18.89 %	12768781	18.58 %	12198001	18.31 %
Unique match	53873689	81.80 %	57434680	83.58 %	55751167	83.69 %
Multi-position match	2434925	3.70 %	2519173	3.67 %	2600421	3.90 %
Total Unmapped Reads	9548234	14.50 %	8763557	12.75 %	8266462	12.41 %

**Table 3:** Classes of RNAs detected by RNA-seq

	Total	Protein coding	pre-tRNA	rRNA	snRNA	snoRNA	miRNA	Other RNA	Pseudogene	TE
Chr1-5	23,250	21,497/27206^a^	0/631	2/4	13/13	18/71	66/177	339/394	368/924	947/3903
ATMG	126	121/122	2/21	3/3	0	0	0	0	0	0
ATCG	96	87/88	1/37	8/8	0	0	0	0	0	0
Total expressed genes	23472	21705/27416	3/689	13/15	13/13	18/71	66/177	339/394	368/924	947/3903

^a^ Numbers in denominator are the total gene number of each type of RNAs annotated in TAIR 10.0 database (http://www.arabidopsis.org/portals/genAnnotation/genome_snapshot.jsp)

In order to distinguish the homologous transcripts derived from the nucleus and from the organelles, clean reads were mapped to the Arabidopsis Col-0 mitochondrion-encoded gene set and the chloroplast-encoded gene set, respectively. The results showed that 96 and 126 transcripts encoded by chloroplasts and mitochondria were detected, respectively (Additional files 2 and 3). The average RPKM for mapped nuclear, chloroplast and mitochondrial genes are 14.3, 11040.2 and 155.3, respectively. It is important to note that each leaf cell contains only one nuclear genome but contains approximately 100 chloroplasts, hundreds of mitochondria and each chloroplast and mitochondrion contain a few genomes. This explains the high RPKM of transcripts encoded by both organellar genomes.

**Fig. 1:**
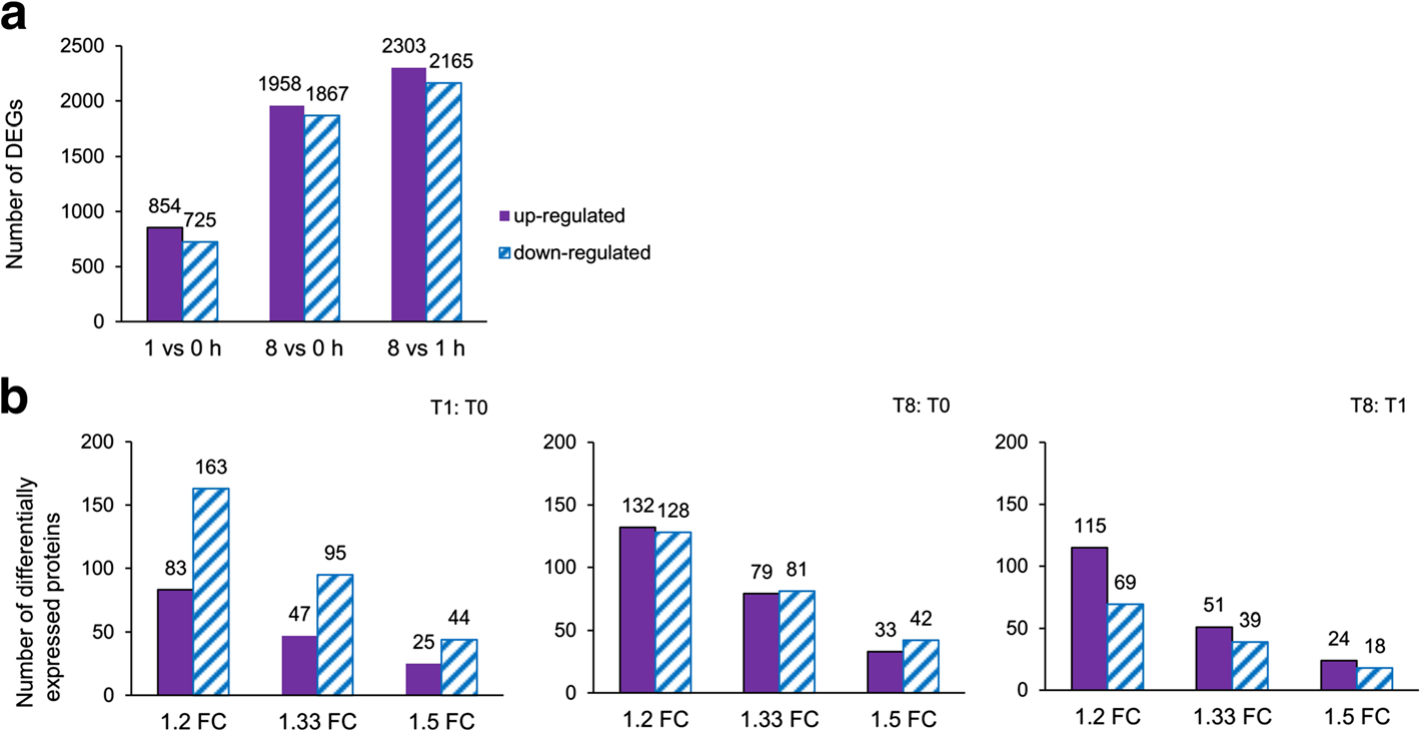
**a** Differentially Expressed Genes were shown in different groups. FDR ≤ 0.001, *P* < 0.01 and 2 FC. **b** Differentially expressed proteins in different groups. FDR < 0.001, *P* < 0.05 and 1.2, 1.33 and 1.5 FC were presented

More transcripts were significantly changed at T8 than at T1 (Fig. 1). The differentially expressed genes (DEGs) at T1 and T0 represented those genes that are immediately responded to illumination, whereas the DEGs between T8 and T1 represented the genes that were indirectly affected by illumination, possibly due to metabolic changes (e.g. ATP, sugars, etc). Most of the differentially expressed genes (log2 ratio ≥ 1 or ≤ -1 and *P*-value < 0.05) were nuclear-encoded or encoded by the mitochondrial genome but none was encoded by the chloroplast genome (Additional file 4).

Alternative splicing (AS) is another area where RNA-sequencing data can provide new information. Generally, there are seven frequent types of AS namely exon skipping (ES), intron retention (IR), alternative 5’ splicing site (A5SS), alternative 3’ splicing site (A3SS), alternative first exon (AFE), alternative last exon (ALE) and mutually exclusive exon (MXE) [11]. To date, 5,885 protein-coding genes in the TAIR 10 database have been documented to exhibit alternative splicing (http://www.arabidopsis.org/portals/genAnnotation/genome_snapshot.jsp). This phenomena has been documented to be affected by time of day [12], environmental conditions [13], and stresses [14]. Our data showed that the most abundant alternative splicing sites were distributed in the type of intron retention and alternative 3’ splicing (Additional file 5). Novel transcripts were also discovered from our samples (Additional file 6). More novel transcripts were detected at time point T1 than were detected at the other time points, however, whether these putative novel transcripts are genuine transcripts remains to be validated in future studies.

Some photosystem transcripts, including *PQL1*, *PQL2*, *ferredoxin1*, *Cyt c6a*, *FdC2*, *Lhca3*, *Lhcb2.3* and *Lhcb4.2* were validated by Quantitative reverse transcriptase PCR (qRT-PCR) using the same total RNA from deep sequencing. The mRNA abundance of selected genes at T0 was adjusted to 1. The ratios of transcript abundance of T1: T0 and T8: T0 were statistically analyzed. The results revealed that all the selected transcripts were consistent between RNA-sequencing and qRT-PCR (Additional file 7).

### Proteomics studies

After strong cation-exchange (SCX) run, fractions were collected at every minute and finally 80 fractions were combined into 9 fractions for LC/MS/MS analysis. The profiles of SCX separation fractions are presented in Additional file 8. Spectra, peptide and protein identification were performed using ProteinPilot software. Results of identified proteins, peptides and spectra with different false discovery rate (FDR) thresholds are presented in Additional file 9A. In total, 2,689 proteins, 19,381 peptides and 81,481 spectra were identified with 95 % confidence in local FDR. 2,872 proteins, 20,343 peptides and 91,147 spectra were identified with 99 % global FDR. 99.9 % confidence happened at local FDR and 88.8 % confidence in global FDR from fit with threshold of 1 % at protein level (Additional file 9B). 2,342 total proteins with 2 or more peptides were identified (Additional files 2, 3 and 10). The number of differentially expressed proteins in different groups were statistically analyzed (*p* < 0.05) and shown in Fig. 1b. Western blotting were carried out to validate the results of proteomics (Additional file 11).

### Effects of light on the transcription and translation of chloroplast genome

Out of 88 chloroplast protein coding genes (TAIR 10.0), 87 CDS were detected in our RNA-seq data (Additional file 2). Since Arabidopsis chromosome genome contains two inverted repeats (ATCG00830-ATCG00900 and ATCG1240-ATCG1310), the reads mapped to these regions were counted twice. By contrast, ProteinPilot only assigned unused peptides to proteins and each peptide is only assigned to one protein. For the repeat, we have to manually copy the proteomics data obtained for one repeat to the other. The transcription levels of most genes were not significantly affected by light (1.5 fold change (FC) cut off, *p* < 0.05). Nonetheless, significant changes could be observed in the respective protein profiles. Out of the 60 chloroplast proteins detected in isobartic tags for relative and absolute quantitation (iTRAQ) experiment, the abundances of only one protein (rps 11) and three proteins (atpE, petA, rpoA) were up- or down-regulated at T1: T0, respectively, whereas the abundances of eight and seven proteins were up-regulated and down-regulated at T8: T0, respectively. All eight up-regulated proteins are ribosomal proteins (rps7.1, rps7.2, rps11, rps18, rpl20, rpl23.1, rpl23.2, rpl32). By contrast, five of the seven down-regulated proteins (psaA, psaB, psbA, psbC, psbD, atpI, ycf4) at T8 are core proteins of photosystem I (psaA/psaB) and photosystem II (psbA, psbC, psbD). No correlation between transcriptome and proteome could be observed.

### Effects of light on the transcription and translation of mitochondrial genome

Among the 122 mitochondria CDS in the Arabidopsis database (TAIR 10), 121 mitochondria CDS transcripts and 11 proteins were detected in the RNA-sequencing and iTRAQ data, respectively. In contrast to the plastid transcripts, the abundance of many mitochondrial transcripts showed significant increase or decrease upon illumination, of which almost all of them encode proteins of uncharacterized functions. By contrast, the abundances of very few proteins were affected by illumination (Additional file 3).

Counterpart homologs of 38 mitochondria-encoded genes are also found in a single syntenic block in nuclear chromosome 2 (AT2G07671.1 ~ AT2G07777.1) with several minor inversions. Strikingly, the orthologous gene pairs between the intercompartmental collinear blocks are extremely similar with most of them being exactly the same with 100 % amino acid identity. For these homologous genes, caution must be taken when gene expression (RPKM) and protein abundance levels are interpreted.

### Effects of illumination on the transcription and translation of photosystems

**Fig. 2:**
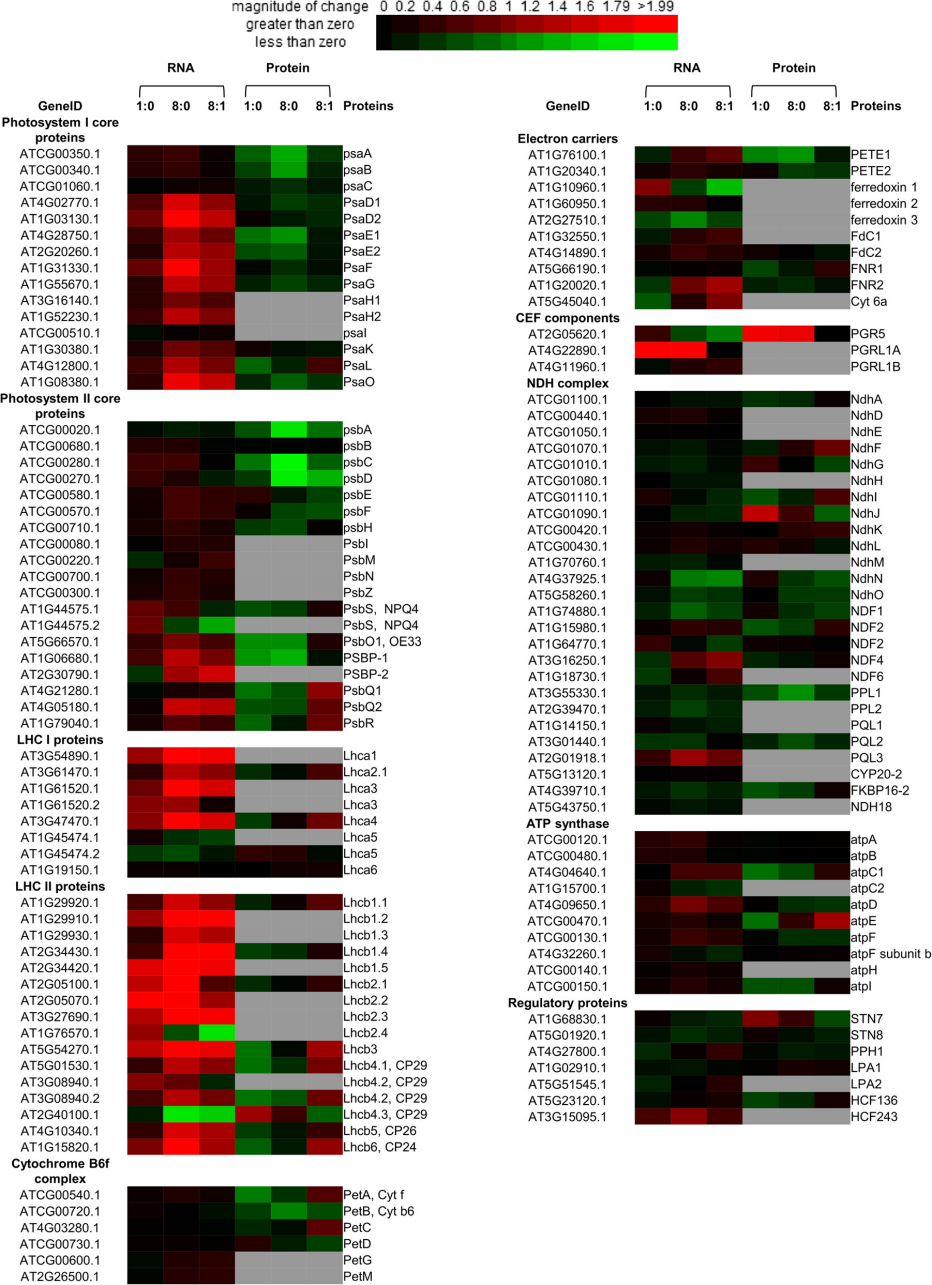
Heatmap of transcription and translation profiles of chloroplast photosystems at different time points. Each value was calculated by log2 ratio and colors were scaled per row with up-regulated in red and down-regulated in green. Missing data were represented by grey boxes. Heatmap was generated from http://bbc.botany.utoronto.ca/ntools/cgi-bin/ntools_heatmapper_plus.cgi. Ratios of (T1:T0, T8:T1 and T8:T0) are compared between each two time points

Comparing to the transcript levels in dark, most genes involved in photosynthesis were significantly up-regulated upon illumination (Additional file 12). While mRNA transcriptions of photosystems I and II components encoded by the chloroplast genome were not significantly changed, the transcription of PSI and PSII components encoded by the nuclear genome were significantly up-regulated. This was also true for LHC (*Lhca1-4*) and LHCII (*Lhcb1-6*) genes, which are encoded by the nuclear genome. By contrast, the transcriptions of NDH complexes and Cyt b6f complexes as well as *Lhca5* and *Lhca6* were not significantly altered. The transcription of some soluble electron carriers, including *PETE1*, *Fd1*, *cyt c6a* and *FNR2* also changed significantly. For some genes, the increase in transcript abundance happened within an hour (T1), but for most genes longer time (T8) was required (Additional file 12). Regarding protein abundance, while the levels of RuBisCo large subunit (ATCG00490.1) and ATP synthase subunit (ATCG00480.1) remained constant (ratio = 1.00), the abundances of some electron transport proteins (PsaE1, PsaE2, PetA, PETE1, FNR1), components of oxygen-evolving complex (OEC) (PsbO1, PsbP1) and ATP synthase subunits C1 and E were down-regulated at T1: T0. Furthermore following prolonged illumination (T8), the protein abundance of some components of photosystem I (PsaA, PsaB, PsaE1, PsaE2), photosystem II (PsbA, PsbC, PsbD), OEC (PsbO1, PsbP1) were down-regulated (Fig. 2).

In summary, while many transcripts were significantly up-regulated upon illumination, protein abundances did not increase in most cases, suggesting that other factors, such as translational control and protein turnover may also affect protein abundance.

### Effects of illumination on transcription and translation of redox proteins and enzymes of the central carbon metabolism

Upon illumination of C_3_ plants, ATP and NADPH are generated from the photosystem. Utilizing the ATP and NADPH, CO_2_ is fixed to three-carbon compounds through the Calvin–Benson–Bassham (CBB) cycle. These C_3_ compounds are used to synthesize starch in the plastid or exported to the cytosol for sucrose synthesis or ATP generation through glycolysis, TCA cycle and respiration in mitochondria. The changes in transcript and protein abundances of the above pathways upon illumination are shown in Additional files 13, 14, 15, 16, 17 and 18. Surplus electrons from LEF can be passed to Fd-dependent enzymes for nitrogen and sulfur assimilation, or to thioredoxin (through FTR) and NADPH (through FNR). A few proteins of the CBB cycle were found to be significantly reduced at T1 and T8 (Additional file 13). Metabolite profiling verified that the amount of sucrose was significantly increased at T1 but not at T8 (Table 4). The amount of SPS protein (AT5G20280.1), the rate-limiting enzyme of sucrose synthesis, increased at T1 and T8, without substantial changes in mRNA transcription (Additional file 14). For enzymes in glycolysis (Additional file 15) and TCA cycle (Additional file 16), the protein abundance of most enzymes did not show significantly changes. Regarding the enzymes in respiratory chains, their mRNA transcripts were mostly unaffected by illumination. The changes in transcript and protein abundances of redox proteins upon illumination are shown in Additional file 17. For protein abundance, only a few components of Complex III (AT4G32470.1, AT5G05370.1 and AT5G40810.1) increased significantly at T1, whereas only one component of Complex I (AT2G27730.1) and one component of Complex II (AT5G40650.1) decreased significantly at T1 (Additional file 18).

**Table 4:** Metabolomic data of 20-d-old WT Arabidopsis leaves at T0, T1 and T8 after illumination

	T0	T1	T8
Amino acids			
Alanine	1.00 ± 0.04	1.22 ± 0.06*	1.48 ± 0.12*
Alanine	1.00 ± 0.05	0.73 ± 0.03*	1.92 ± 0.14*
Asparagine	1.00 ± 0.13	0.61 ± 0.07*	0.51 ± 0.05*
Aspartic acid	1.00 ± 0.05	0.74 ± 0.07*	1.07 ± 0.14
Butyric acid, 4-amino	1.00 ± 0.06	0.62 ± 0.10*	0.59 ± 0.09*
Cysteine	1.00 ± 0.16	0.83 ± 0.10	0.94 ± 0.11
Glutamic acid	1.00 ± 0.05	0.94 ± 0.05	0.67 ± 0.06*
Glutamine	1.00 ± 0.07	0.66 ± 0.06*	0.76 ± 0.10
Isoleucine	1.00 ± 0.04	1.31 ± 0.08*	1.73 ± 0.13*
Lysine	1.00 ± 0.05	0.71 ± 0.05*	0.89 ± 0.08
Methionine	1.00 ± 0.05	2.29 ± 0.14*	2.30 ± 0.18*
Phenylalanine	1.00 ± 0.08	1.67 ± 0.05*	1.24 ± 0.07
Proline	1.00 ± 0.05	2.01 ± 0.09*	1.53 ± 0.12*
Pyroglutamic acid	1.00 ± 0.03	0.71 ± 0.04*	0.63 ± 0.07*
Serine	1.00 ± 0.05	4.23 ± 0.21*	7.17 ± 0.47*
Threonine	1.00 ± 0.03	1.71 ± 0.09*	2.69 ± 0.15*
Valine	1.00 ± 0.07	1.03 ± 0.04	1.36 ± 0.08*
Organic acids			
Benzoic acid	1.00 ± 0.04	1.13 ± 0.08	1.08 ± 0.05
Citric acid	1.00 ± 0.29	1.30 ± 0.59	0.95 ± 0.35
Dehydroascorbic acid	1.00 ± 0.09	1.43 ± 0.41	1.46 ± 0.34
Fumaric acid	1.00 ± 0.05	1.56 ± 0.23	3.13 ± 0.27*
Galactonic acid	1.00 ± 0.05	0.85 ± 0.04	0.99 ± 0.08
Glyceric acid	1.00 ± 0.05	2.11 ± 0.17*	4.99 ± 0.36*
Lactic acid	1.00 ± 0.12	0.94 ± 0.09	1.01 ± 0.17
Malic acid	1.00 ± 0.05	1.08 ± 0.20	2.02 ± 0.22*
Nicotinic acid	1.00 ± 0.06	1.00 ± 0.05	0.93 ± 0.05
Phosphoric acid	1.00 ± 0.26	0.98 ± 0.30	0.96 ± 0.30
Succinic acid	1.00 ± 0.03	0.84 ± 0.08	0.53 ± 0.05*
Sugars			
Lyxose	1.00 ± 0.06	1.13 ± 0.02	1.32 ± 0.05*
Fructose	1.00 ± 0.02	2.59 ± 0.14*	0.81 ± 0.04*
Fucose	1.00 ± 0.05	1.00 ± 0.04	0.93 ± 0.04
Glucose	1.00 ± 0.08	1.28 ± 0.06*	0.61 ± 0.04*
Glucose, 1,6-anhydro	1.00 ± 0.08	1.63 ± 0.08*	1.67 ± 0.12*
Inositol	1.00 ± 0.04	0.87 ± 0.04	0.73 ± 0.04*
Sorbitol	1.00 ± 0.05	0.91 ± 0.05	1.09 ± 0.09
Sucrose	1.00 ± 0.05	1.51 ± 0.14*	1.27 ± 0.17
Others			
Mannopyranoside, 1-O-methyl-	1.00 ± 0.07	1.09 ± 0.03	0.94 ± 0.07
Ornithine	1.00 ± 0.10	1.84 ± 0.09*	1.30 ± 0.15
Putrescine	1.00 ± 0.09	1.12 ± 0.07	1.57 ± 0.08*
Spermidine	1.00 ± 0.16	2.47 ± 0.18*	4.11 ± 0.45*
Threitol	1.00 ± 0.05	0.78 ± 0.05*	0.78 ± 0.05

Data are normalized to the mean response calculated for the time point (T0) of each measured batch. Values are presented as the mean ± SE of 6 biological determinations. Asterisks at T1 and T8 indicate values significantly different from T0, as calculated by *t* test (increase and decrease) with *p*-value < 0.01

### Metabolomic and pathway activity analyses

**Fig. 3:**
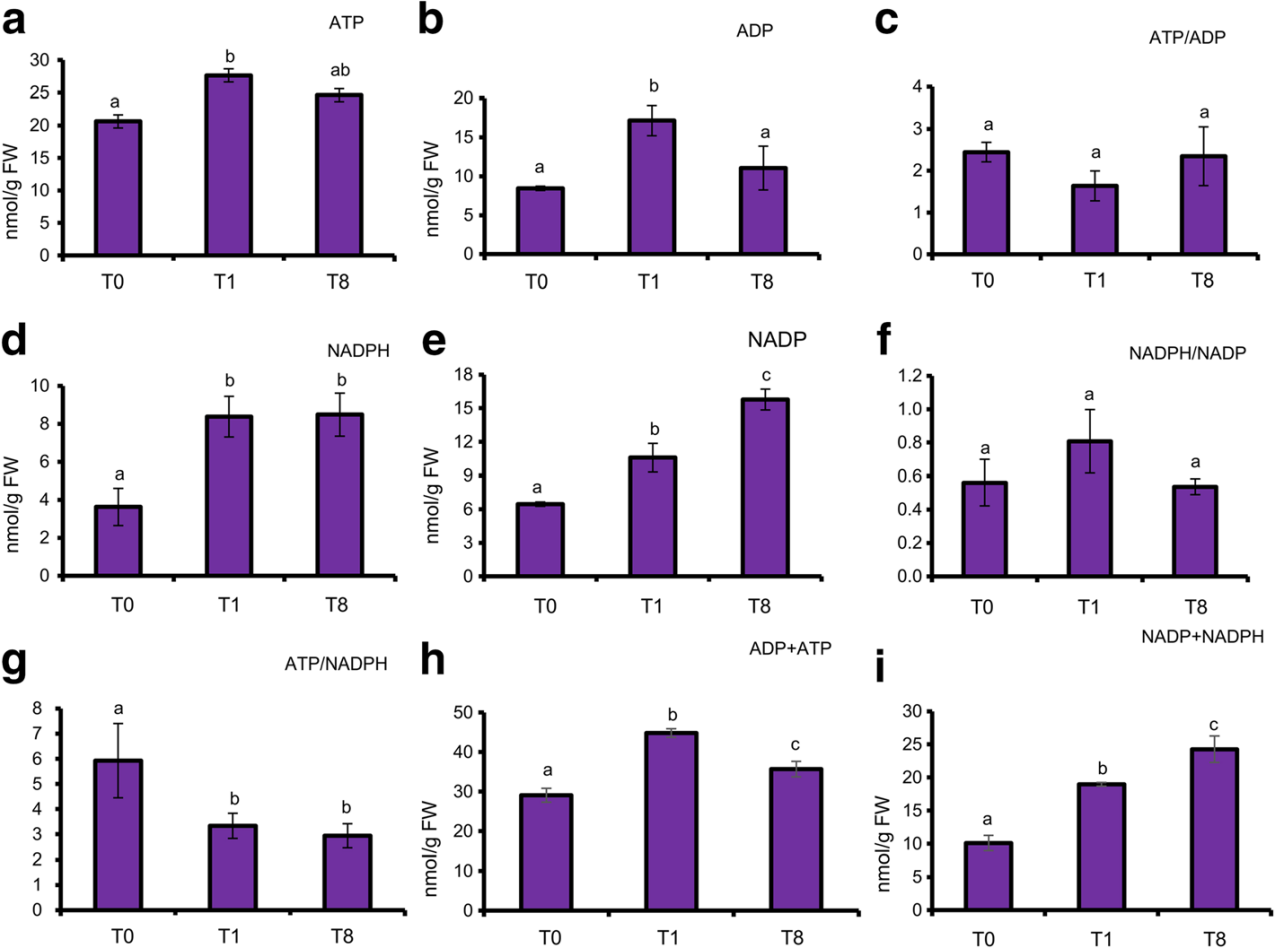
Metabolites were measured from 20-d-old Arabidopsis leaves of WT at T0, T1 and T8. **a** ATP, **b** ADP, **c** ATP/ADP, **d** NADPH, **e** NADP^+^, **f** NADPH/NADP^+^, **g** ATP/NADPH, **h** ADP + ATP and **i** NADP^+^+NADPH were presented respectively. Data were expressed as means with ± SD of three biological replicates. Statistical differences (*P* < 0.05) in the same column for each line were based on one-way ANOVA analysis followed by Tukey’s Honestly Significant Differences (HSD) test using statistical program IBM SPSS 19. Within each column, the values marked by different letters (*a*, *b*, *c*) are significantly different (*P* < 0.05). The data were reproducible in at least 3 independent experiments. FW: Fresh weight

The levels of ATP, ADP, NADP^+^ and NADPH in leaves of 20-d-old Arabidopsis plants harvested at different time points of illumination were measured. Compared to the level measured at T0, the ATP content in leaf was significantly higher at T1 but its level dropped slightly after 8 h illumination (Fig. 3). The same trend occurred in ADP levels during dark to light transition. As the levels of both ATP and ADP change in similar extends, the ratio of ATP/ADP was invariant at all three time points. For NADPH, the levels were more than two folds during illumination (T1 and T8) compared with that at the end of night (T0). Since a large amount of NADPH is produced by linear electron flow (LEF) under light condition, it is reasonable that the levels of the metabolites were higher under illumination. As NADPH displayed greater than two fold increase while ATP only had slight increase upon illumination, the ATP/NADPH ratio dropped significantly under illumination. By contrast, the NADPH/NADP^+^ ratios were indifferent between the three time points.

Metabolites measured using a GC-MS platform, including amino acids, organic acids sugars and others are shown in Table 4. While the levels of glucose, fructose, and sucrose significantly increased at T1, the levels of glucose and fructose significantly decreased at T8. Regarding TCA metabolites, the levels of malate and fumarate increased significantly but the level of succinate decreased significantly at T8.

**Fig. 4:**
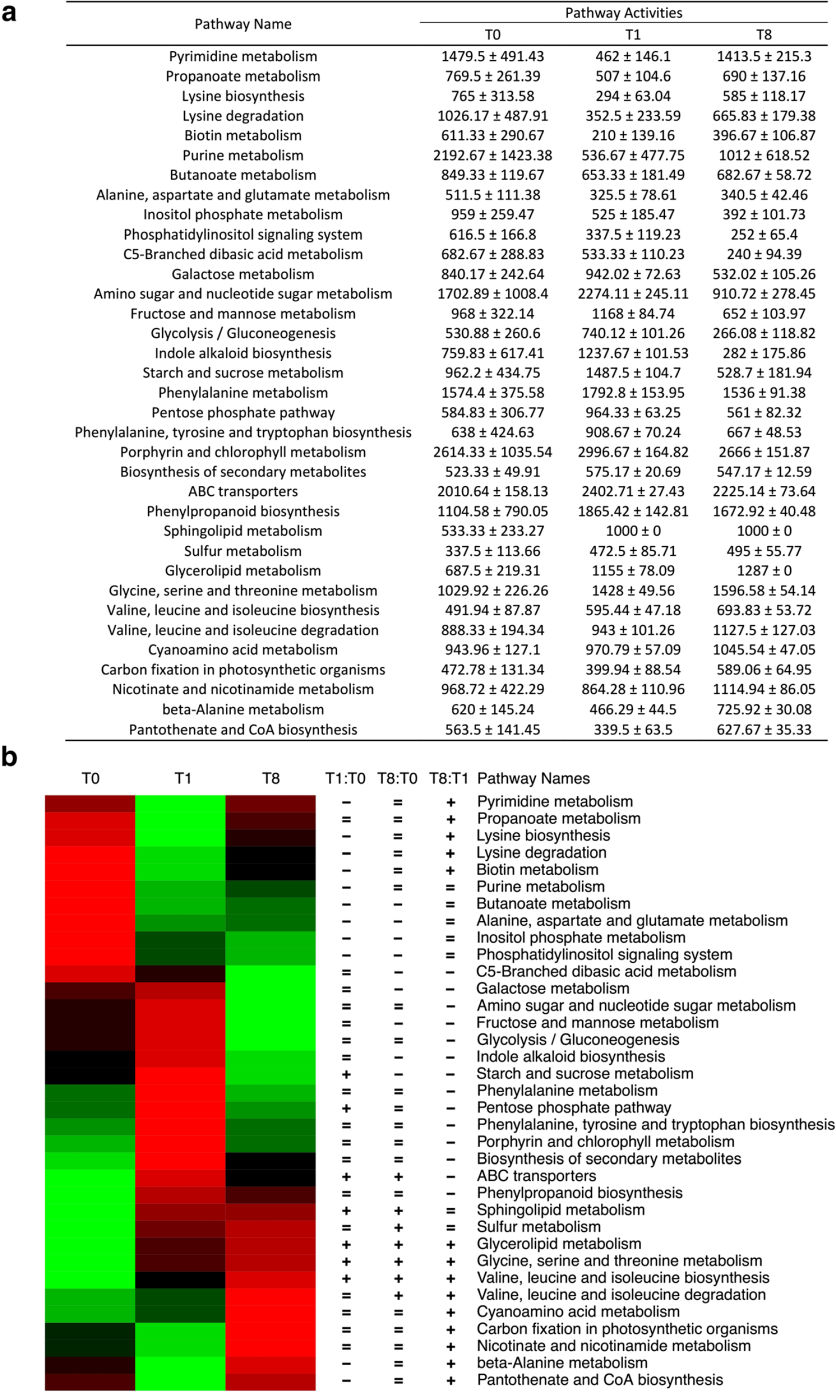
Calculation of pathway activity based on metabolomic data analysis. **a** The Table shows pathways that significantly differentially activity levels were discovered in pairwise comparisons (t-test, *p* < 0.05) between any two of the three time points (T0, T1, T8). **b** Heatmap for significantly different pathway activities. Red represents higher pathway activity while green stands for a less active. Pathway names were marked with three columns of signs, illustrating the significant level of the three pathway-based pairwise comparisons. From left to right, three signs represent the T1:T0, T8:T0 and T8:T1 comparisons, respectively. ‘+’ stands for significantly more active (T1 > T0, T8 > T0 or T8 > T1), ‘-’ stands for significantly less active (T1 < T0, T8 < T0 or T8 < T1), and ‘=’ is for statistically insignificant comparisons. The significance cut-off was set to *p*-value < 0.05

Pathway activities were calculated based on the metabolome data (Additional file 19), using the Pathway Activity Profiling (PAPi) algorithm. In total for 35 pathways significantly different activity levels were discovered in pairwise comparisons (*t*-test, *P* < 0.05) between any two of the three time points (Fig. 4). At T1, the activities of starch and sucrose metabolism, pentose phosphate pathway, valine, leucine and isoleucine synthesis, glycine, serine and threonine metabolism were significantly higher than T0 (T1 > T0) but that of purine, pyrimidine alanine, aspartate, glutamate and lysine metabolisms were significantly lower (T1 < T0). Notably, the pathway activities of major carbon metabolism, including starch and sucrose metabolism, pentose phosphate pathway, glycolysis/gluconeogenesis, galactose, fructose, mannose metabolism, amino sugar and nucleotide sugar metabolism were significantly lower after prolonged illumination (T1 > T8). Interestingly, the pathway activity of the glycerolipid metabolism was significantly increased by time in all three comparisons (T1:T0, T8:T0 and T8:T1). A similar trend was observed for the glycine, serine and threonine metabolism and valine, leucine and isoleucine biosynthesis (Fig. 4).

### Integration of transcriptome and proteome analyses with metabolome-based pathway activity data

**Fig. 5:**
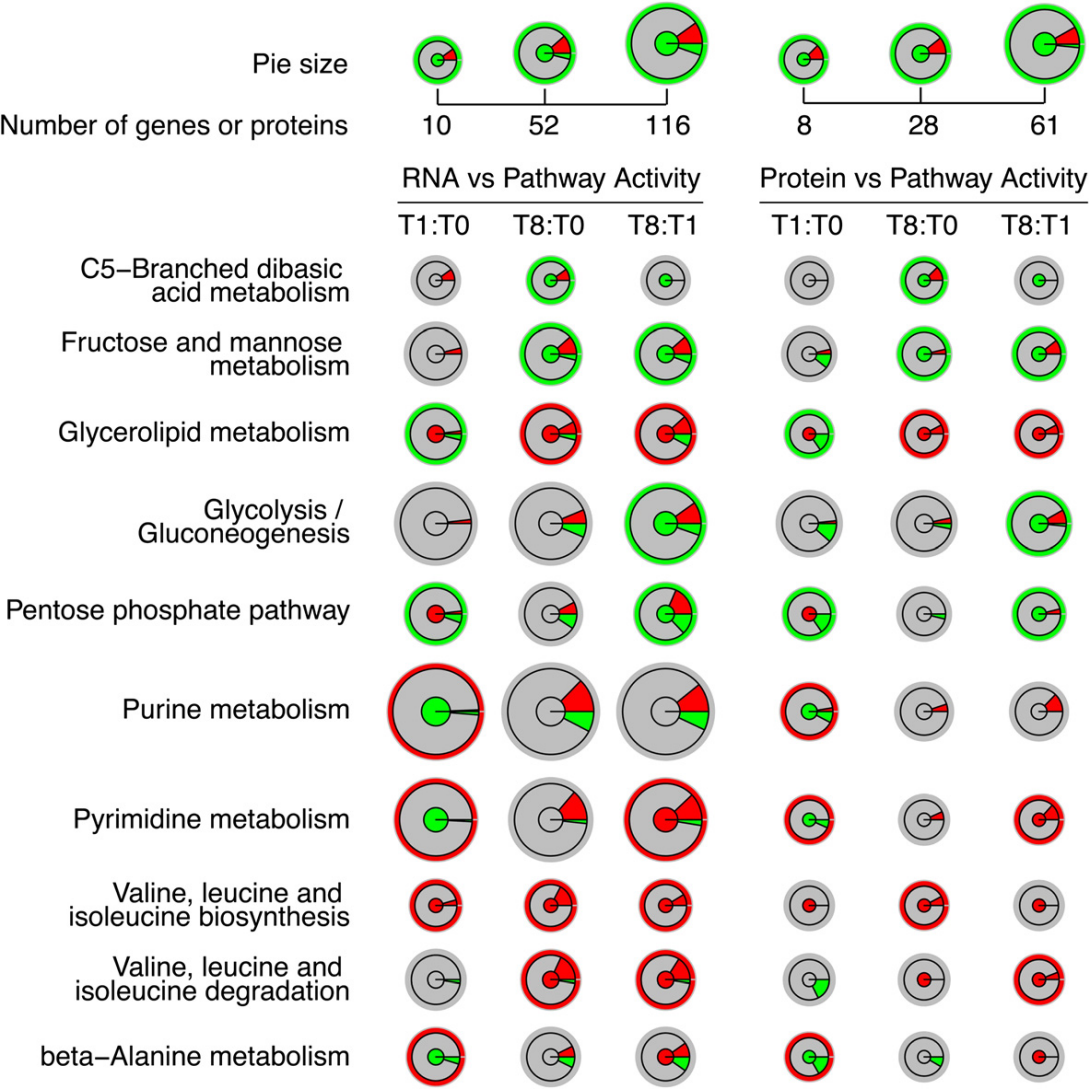
The cases where similar trends in the correlations between metabolome-based pathway activity and RNA or protein level were observed. Three pairwise comparisons based on the time points T0, T1, T8 were included, for RNA and protein levels respectively. The radius represents the volume (total number of genes or proteins) of the target pathway. Inner circle represents the metabolome-based pathway activity; gray: no significant difference in pathway activities; red: significantly more active; green: significantly less active. The intermediate ring stands for ratio of up- or down-regulated genes or proteins from the transcriptome or proteome comparison; gray: genes or proteins not significantly differentially expressed; red: up-regulated genes or proteins; green: down-regulated genes or proteins. The outer thin ring stands for the relationship between metabolome-based pathway activity and predicted RNA or protein level pathway activity; red: positive correlation, green: negative correlation

Differentially expressed genes were mapped to >100 pathways in KEGG database for *Arabidopsis thaliana*. We calculated the numbers of all up- or down-regulated genes for all pathways in the three pairwise comparisons between any two of the three time points (Additional file 20). The average ratio of significance denoted as the number of significant genes divided by the total number of genes in the pathway was ~16 %. Photosynthesis - antenna proteins, flavone and flavonol biosynthesis and brassinosteroid biosynthesis were the metabolic pathways with the highest number of genes found to significantly alter their expression levels (70, 67, and 38 %, respectively). We hypothesized that the pathway activity should in general be higher when there are more up- than down-regulated genes, and this information, theoretically, should to some degree be correlated with the metabolome-based pathway activities. As shown in Fig. 5, there are several pathways in which this correlation could be observed. In valine, leucine and isoleucine biosynthesis the metabolome-based pathway activity was significantly increased in all pairwise comparisons. All the genes that were found significantly differentially expressed were up-regulated (Fig. 5). A similar trend was observed in the beta-alanine (T1:T0) and pyrimidine metabolism (T1:T0). There were also cases that a correlation between metabolome and RNA data could still be observed even though both up- and down-regulated genes were retrieved from the pairwise comparisons. For the pathways valine, leucine and isoleucine degradation (T8:T1 and T8:T0), pyrimidine metabolism (T8:T1) and glycerolipid metabolism (T8:T0 and T8:T1), a higher ratio of up- or down-regulated genes in the pathway resulted to an increased or decreased, respectively, metabolome-based pathway activity (Fig. 5). What was also interesting is that for all the aforementioned pathways the central dogma of biology was observed: the ratio of proteins with increased or decreased abundance was correlated to the ratio of up- or down-regulated genes, respectively, and of course further correlated with the metabolome-based pathway activities (Fig. 5).

In contrast, there were also pathways showing a negative correlation between metabolome-based activities and ratios of up- and down-regulated genes/proteins (Fig. 5); the C5-branched dibasic acid metabolism (T8:T0), fructose and mannose metabolism (T8:T0 and T8:T1), glycerolipid metabolism (T1:T0), glycolysis/gluconeogenesis (T8:T1) and pentose phosphate pathway (T1:T0 and T8:T1) are such pathways where the changes in the gene expression are depicted in the protein abundance but are reversed in the metabolic activities of these pathways. Other notable pathways where the hypothesis of a correlation between the direction (up- or down-regulation) of the majority of the significantly altered genes in a pathway and the metabolome-based pathway activity did not stand true were the alanine, aspartate and glutamate metabolism and lysine degradation (Additional file 20). All the genes that were found significantly differentially expressed in the T1:T0 comparison of alanine, aspartate and glutamate metabolism and lysine degradation were up-regulated; nevertheless the metabolome-based pathway activity was decreased. However, in both pathways a correlation between protein abundance and metabolome-based pathway activity was observed; the level of the proteins found differentially expressed in the two pathways were lower in the T1 compared to T0. The T8:T1 comparison of the carbon fixation pathway was another case were the metabolome-based activity (increased) was positive correlated with the proteome (higher number of proteins with increased abundance) but not the transcriptome data (Additional file 20).

## Discussion

Chloroplasts and mitochondria orchestrate to generate energy for various biochemical reactions [15]. Chloroplasts produce reducing power, ATP and triose phosphates and mitochondria consume reducing power and carbohydrates produced by chloroplasts to generate ATP [15, 16]. The mitochondrial respiratory chain also plays an important role in maintaining the redox balance in plant cells [17]. While photosynthetic oxygen evolution, which generally reflects the combined activities of chloroplasts and mitochondria, responds to illumination within a minute [18], the transcriptional and translational responses induced by illumination usually take longer time. The energy-generating electron-transfer chains in chloroplasts (photosystems) and mitochondria (respiratory complexes) are both encoded by the nuclear genome and the organellar genomes [19]. Hence, transcription activities of chloroplast and mitochondrial genomes are also critical for investigating plant energy metabolic changes during dark to illumination conversion. RNA-seq by sequencing total RNA without ribosomal RNAs allowed us to obtain information on transcripts encoded by the chloroplast and mitochondrial genomes. Our method thus depicts a more complete picture of the changes in abundances of RNA transcripts encoding the photosystem (Fig. 2) and respiratory complexes (Additional file 18). This study also identified 2,342 proteins (no less than 2 peptides) encoded by both nuclear and organellar genomes and examine the changes in their abundance upon illumination.

Photosynthesis is the ultimate source of energy for plants. In this study, we followed the changes in metabolites, mRNA levels and protein abundance of the leaves of Arabidopsis after illumination. Comparing T8 to T0, RNA-seq data (Fig. 2) revealed that the transcription of the genes of all *LHCI* (*A1-4*), *LHCII* (*B1-6*), OEC complexes (*psbO*, *P*, *Q*), were up-regulated (FC > ±1.5, FDR < 0.001). This is also true for the PSI and PSII components encoded by the nuclear genome. When comparing T1 to T0, only the transcription of some genes of *LHCI* (*A1 - 4*), *LHCII* (*B1*, *B2*, *B3* and *B6*), *psaD1/D2, psaF* were up-regulated (FC > 1.5, FDR < 0.001). By contrast, the transcription of genes encoding cytochrome b6f and ATP synthase (except *atpD*, which was up-regulated at T8 vs T0), were not significantly changed. The above RNA-sequencing data suggest that the transcriptions of genes encoding different functional complexes in the photosystem are differentially regulated but members of the same complex are co-regulated with each other. While the transcription of the genes described above were significantly up-regulated at T1 and T8, their protein abundances did not alter significantly. By contrast, the protein abundance of PsaE1/E2, PsbO, PsbP, Cyt f, PETE1 and FNR1 were down-regulated at T1, whereas PsaA/B and PsaE1/E2 of PSI, PsbA, PsbC, PsbD of PSII, PsbO and PsbP of OEC and PETE1 were down-regulated at T8. Two proteins had abundance decreased at T1, but increased at T8, namely cytochrome f and PsaL, the docking site of LHCII on PSI. It should be noted that both PsaA and PsaB [20], and PsbC and PsbD [21] are transcribed as di-cistronic transcripts. While their RNA levels were steady across the three time points, their co-downregulation in protein abundance implies that the translational efficiency of the dicistronic transcripts might be compromised upon prolonged illumination.

Proteomics studies of dark-grown etiolated rice seedlings revealed that the protein abundances of major photosystem proteins increased significantly upon 2-3 h illumination [22]. This is physiologically relevant during the greening process of plastids. By contrast, our proteomics data showed that protein abundance of some photosystem proteins in mature Arabidopsis leaves decreased following 8-h of illumination. Why the protein abundances of the core proteins of PSI (PsaA/B), PSII (PsbA/C/D) and OEC (PsbO/P) decreased at T8? The purpose might be to reduce the harvest of light energy and the overproduction of electrons after prolonged illumination, which may cause damage to the photosystem. Arabidopsis chloroplasts contain at least six Deg proteases [23], of which Deg1 was reported to degrade photosystem core proteins D1/D2 (PsbA/D) [24]. These data indicate that the protein abundances of photosystem components are likely to be subject to complex and versatile regulation.

The PSI and PSII protein components are encoded by both nuclear and plastid genomes. Our RNA-sequencing data showed that while the transcription of the nuclear genes was up-regulated at T8, the transcription of the genes encoded by the plastid genome did not change significantly (Additional file 12). The transcription of chloroplast genome is carried out by PEP (Plastid-Encoded Plastid RNA polymerase) and NEP (Nuclear-Encoded Plastid RNA polymerase). PEP is involved in the transcription of tRNAs and a number of photosynthesis genes (*psaA*, *psbA-D, psbEFLJ*) under the control of six nuclear-encoded Sigma factors [25, 26]. NEP is involved in the transcription of a number of housekeeping genes (e.g. *accD, atpB, rpoB*) under the control of different NEP promoters [25]. Nonetheless, the transcription of some chloroplast genes (*atpA, clpP, rpl33, rrn5, rrn16* and *rrn23*) are controlled by both PEP and NEP [26]. In the transcription data of chloroplast genome (Additional file 2), the transcript abundance did not change much after 1 h of illumination. Only the transcription of a tRNA (*TRNS.2*) was increased by 1.6x. After 8 h of illumination, the transcript abundances of *TRNS.2*, two psb genes (*psbL* and *psbJ*) and a few ribosomal proteins (*rps12a, rps12b rps12c, rpl20*) were significantly increased, and that of *rrn16* and *rrn23* were significantly decreased. The transcription of the two *psb* genes and *TRNS.2* were controlled by SIG1 and SIG2, respectively, and the transcription of ribosomal RNA (*rrn*) operon are transcribed by both PEP and NEP [27]. Hence, illumination affects the transcription of these chloroplast genes through both PEP and NEP and the regulation is complex. Mitochondrial transcription is carried out by nuclear-encoded RNA polymerase of the T3/7 phage (RpoT) and there are 2 RpoT targeted to mitochondria (RpoTm and RpoTmp) in Eudicots [28]. RpoTm was proposed to be the basic RpoT for the transcription of most mitochondrial genes and RpoTmp plays a specific role in the transcription of *cox1, ccmC, matR, nad1, nad2*, *nad6* and *rps4* [29]. Upon illumination, the transcript abundance of 17 and 13 mitochondrial transcripts were enhanced (FC > = 1.5) at T = 1 and T = 8, respectively. Most of them (*orf*) encode for uncharacterized proteins, except for *matR* (T = 1) and *rpl5*, *rpl16* and *ccb206* (T = 8) transcripts. Hence, illumination also affect the transcription of these mitochondrial genes through RpoT and RpoTmp and the regulation is complex.

While the transcription of most chloroplast genes is steady upon illumination, the protein abundance of eight and seven proteins encoded by the chloroplast genome were significantly up- or down-regulated (FC > ±1.5, *p* < 0.05) (Additional file 12). This implies that the abundance of these proteins could be regulated by differential translational regulation or protein degradation [23, 24]. This was also reported for proteins translated by the mitoribosomes [30].

Photosynthesis is the ultimate source of reducing power and energy to plants. During photosynthesis, electrons are extracted from water molecules and passed to ferredoxin (Fd) via LEF. Reduced Fd could then pass the electrons to NADPH by (ferredoxin NADP^+^-reductase, FNR) or thioredoxin (via ferredoxin thioredoxin reductase, FTR) and to reduce sulfite and nitrite by ferredoxin-nitrite-reductase and sulfite reductase, respectively. All these reducing power-consuming processes are driven by light. This explains why under illumination the levels of NADPH significantly increased at both T1 and T8 (Fig. 3). While photosynthesis also generates ATP, the level of ATP only slightly increased at T1 but insignificant at T8 (Fig. 3). Three ATP molecules and two NADPH molecules are required to fix one CO_2_ molecule in the CBB cycle. The LEF generates ATP and NADPH at a ratio of 1.29, and the shortfall in ATP has to be fulfilled by other mechanisms such as cyclic electron flow [31] or from mitochondria [17]. Alternatively, excess reducing power generated from the LEF has to be exported to cytosol and consumed by mitochondria to produce ATP. The more significant increase in NADPH content but to a much lesser extent in ATP content in leaves upon illumination (Fig. 3) reflects that the demand of ATP for anabolic processes (e.g. starch, sucrose, cell wall syntheses) is very high during photosynthesis and that the reducing power generated from the photosystems is more than adequate. Excess reducing power from photosynthesis can be exported from the chloroplasts by the malate valve, and eventually converted into NADH for ATP production at the mitochondria [16]. Malate content in leaves increased 2-fold under prolonged illumination (Table 4), perhaps reflecting its role as a substrate of the mitochondrial respiratory chain for ATP production [32]. Flux-balanced analysis predicted that mitochondrial and chloroplast ATP synthases contribute 18 and 82 % of ATP synthesis when the light intensity was between 180 to 280 μmol m^−2^ s^−1^ in C3 plants [33]. Whether the ATP produced by chloroplast ATP synthase is adequate for carbon fixation is an interesting question. Oligomycin treatment of barley protoplasts significantly reduced the ATP levels in mitochondria and cytosol, but not the ATP level in chloroplasts, indicating the importance of mitochondria in supplying ATP to cytosol [16].

The carbon fixed by photosynthesis is exported to the cytosol as dihydroxyacetone phosphate (DHAP). DHAP can serve two major purposes, anabolism (sucrose or cell wall synthesis) or the generation of ATP through the glycolysis (Additional file 15), the TCA cycle (Additional file 16) and mitochondria respiration (Additional file 18). The TCA cycle operates in cyclic mode in dark (to produce NADH/FADH_2_ and ATP) when TCA is the major source of ATP and in non-cyclic mode (to produce skeletons for amino acid synthesis) under illumination [32, 34]. Short-term (5 s – 60 mins) ^13^CO_2_ labeling of Arabidopsis rosette leaves showed that fixed carbon is rapidly incorporated into CBB intermediates and ADP-Glu (for starch synthesis), followed by metabolites for sucrose synthesis and photorespiration, but very slowly or even negligibly into TCA intermediates [35]. While cyclic TCA is reduced in light, it is interesting to note that not many enzymes in glycolysis (Additional file 15) and TCA cycle (Additional file 16) changed in protein abundance as many of these enzymes are regulated allosterically by the ATP/ADP ratio [36].

Traditionally, it was believed that protein expression levels are determined by transcript expression levels. However, our data showed that this is not always true (Additional file 21). This discrepancy could be due to the following reasons: A fraction of transcribed mRNAs is not translated or is translated at a lower rate [37]. There are two pools of mRNAs in plant cells: free mRNAs and polysomes-associated mRNAs. Light can promote the association of some mRNA to ribosomes and drive their translations. In this case, protein abundance can increase without transcription [38]. Our RNA-seq data only measured the expression levels of steady-state mRNA, which is the sum of free and polysomes-associated mRNAs. Hence, there will be discrepancies between the steady-state mRNAs and protein abundance; (2) Some transcribed mRNA may have a short half-life [39] for example those that are subject to degradation by sRNA-mediated processes [40]. RNA-seq may also sequence partially degraded mRNA, which is not translatable. The half-lives of Arabidopsis mRNA vary from minutes to >24 h. mRNAs with shorter half-lives may translate less protein molecules per mRNA molecule. Our iTRAQ data compared the relative abundance of proteins, which might have been accumulated for hours; (3) Protein abundance is determined by both translation and degradation. Protein abundance may drop despite of a constant level of mRNA. The above reasons can explain the discrepancies between mRNA and protein abundances resulting to the negative correlation that was observed in our study. One of the most interesting examples is the negative correlation between mRNA and protein abundance of the photosystem components (Fig. 2). Such lack of correlation is not only observed for nuclear genes, but also for the genes encoded by the chloroplast genome.

In summary, this study provides integrated datasets on the impacts of light on the transcription and translation of genes encoded by all three genomes of plant cells. The changes of transcriptome and proteome of central metabolisms have been analyzed in details in this report. The datasets will also be useful for researchers interested in secondary metabolisms.

## Conclusion

Since proteins are the agents to carry out biochemical conversions and biological processes and in general, there is a lack of positive correlation between mRNA transcription and protein levels, the understanding of plant physiology in previous reports, which solely employed microarray or mRNA expression data for interpretation, should be carefully interpreted. This study provides leaf omics data on the changes of transcriptome, proteome and metabolite profiles of 20-day-old Arabidopsis leaves upon illumination. The data will provide a database to plant researchers to look up their gene of interests and examine their changes in mRNA and protein abundance in leaf upon 1 and 8 h illumination. This study also provides information on the changes in transcriptome and proteome profiles of chloroplasts and mitochondria upon illumination.

## Methods

### Plant materials and growth conditions

*Arabidopsis thaliana* ecotypes Columbia (Col-0) from TAIR was used in this study. After sterilization, seeds were placed on Murashige and Skoog medium supplemented with 2 % (w/v) sucrose for 10 days and subsequently seedlings were transferred to soil under 16 h light (22 °C)/8 h dark (18 °C) period in growth chamber with a light intensity of 120–150 μmol m^−2^ s^−1^. Leaves of 20 days old Arabidopsis plants were harvested and frozen in liquid nitrogen RNA, protein and metabolite extraction. Leaves were harvested at three different time points: T0 (end of night), T1 (one hour after onset of illumination) and T8 (eight hours after onset of illumination), respectively.

### Transcriptome analysis

Total RNA was extracted from leaves at all three time points and DNA contamination was removed by DNase I (RNeasy Plant Mini Kit, Qiagen, Hong Kong). Ribosomal RNAs were removed from the total RNA by the Ribo-Zero rRNA removal kit for plant leaf (Epicentre, USA) before cDNA library construction. The libraries were sequenced using Illumina HiSeq™2000. After removal of low quality reads, clean reads from three different RNA-seq samples (The three different time points, T0, T1 and T8) were aligned to the Arabidopsis genome. To distinguish the homologous transcripts derived from nucleus and organelles, the clean reads were mapped to the Arabidopsis Col-0 (TAIR10.0) nuclear-encoded CDS gene set, the mitochondria-encoded CDS gene set and the chloroplast-encoded CDS gene set, respectively. The alignment tool is SOAPaligner/SOAP2 (parameters: -m 0 -× 10,000 -s 40 -l 32 -v 5 -r 2 -p 6) [41]. The transcript abundance was estimated by the RPKM (Reads per kilobase transcript per million reads) calculation for each gene in each compartment [42]. RPKM = 109*C/N*L (C is the number of mappable reads that fell onto the genes; N is the total number of maptable reads in the experiment; L is the sum of the genes in base pairs).

Alternative splicing transcripts were analyzed based on SOAPsplice software [43]. For novel transcripts which were not identified in the TAIR10.0 database, the assembled transcripts must meet the following three criteria: (i) the length of the transcript must be more than 180 bp. (ii) the sequencing depth is no less than 2. (iii) the transcripts must be at least 200 bp away from annotated gene. The identified novel transcripts were then distinguished as protein-coding and non-coding RNAs by the use of the Coding Potential Calculator (CPC: http://cpc.cbi.pku.edu.cn/).

### Leaf protein extraction

20-day-old leaves were harvested at T0, T1 and T8. Proteins were extracted and precipitated in 10 % (v/v) TCA/acetone (−20 °C) and the pellet was washed by 10 × volume of 80 % (v/v) methanol/0.1 M NH_4_OAc and precooled 80 % (v/v) acetone, respectively. The pellet was then homogenized in 8 ml SDT buffer (4 % (w/v) SDS, 0.1 M DTT and 0.1 M Tris-Cl pH 8.0) for 2 min. The mixture was heated for 5–10 min at 95 °C followed by centrifuging twice at 16,000 × *g* for 5 min at 4 °C. The supernatant were collected and then 4 × volume of chilled 80 % (v/v) acetone were added to precipitate the proteins at −20 °C overnight. After centrifugation at 2,000 × *g* for 15 min at 4 °C, the pellets were washed with 5–10 × volume of 80 % (v/v) acetone before being air dried. The protein pellet was dissolved with 2 ml urea buffer (6 M urea in 200 mM MOPS)/4 mM CaCl_2_, pH 8.0). Then the mixture was sonicated for 20 cycles of 10 s on and sonication 6 s rest until the pellet was completely dissolved. After centrifugation at 7000 × *g* for 1 min at room temperature, the supernatant were collected and quantified according to the Bradford method [44].

### Reduction, alkylation and trypsin digestion and iTRAQ labeling

The protein extracts were then subjected to iTRAQ labelling to label peptides [45]. Equal amount of protein (100 μg) was reduced by 10 mM dithiothreitol (DTT) and reduced cysteine groups were alkylated by 40 mM iodoacetamide (IAA) in the dark. After alkylation, the mixture was diluted with 4 mM CaCl_2_ to reduce the concentration of urea to less than 2 M. Trypsin was added to digest protein at 1:20 ratio by 1 μg trypsin per 20 μg protein, at 37 °C overnight. After trypsin digestion, the peptides were desalted by C18 SepPak reverse-phase cartridges (Waters, WAT023590, and Ireland). The desalted peptides were then labeled by the 8-plex iTRAQ labeling kit (AB Sciex, USA). For labeling, samples harvested at the 3 time points (T0, T1 and T8) were labeled with 113, 114 and 115, respectively and the second biological replicates were labeled with 117, 118 and 119. All labeled samples (6 tubes) were combined together and the labeled peptides were fractionated by SCX [46]. Chromatographic separation was performed on an Eclipse XDB C18 column (2.1 mm × 150 mm, 5 μm, narrow-bore) (Agilent Technologies, USA) at room temperature with elute A (10 mM H_3_PO_4_/KH_2_PO_4_, 25 % ACN, pH 3.0) and elute B (10 mM H_3_PO_4_/KH_2_PO_4_, 25 % ACN, 350 mM KCl, pH 3.0). Fractions collected from 15–40 mins were combined into 9 fractions for LC/MS/MS analysis (TripleTOF 5600 system, AB SCIEX, USA). Three technical replicates were run. MS data were acquired using a TripleTOF 5600 system fitted with a Nanospray III source (AB SCIEX, USA). The parameters in this experiment were listed below: ion spray voltage (2.2 kV); curtain gas (20 psi); nebulizer gas (6 psi); interface heater temperature (150 °C). For IDA, full scans were acquired at no more than 250 ms over the range *m/z* 400-1250, followed by MS/MS scans of the 20 most abundant peaks that exceeded 125 counts per second and carried a charge between +2 to +5 in the range *m/z* 100-1500 [46]. MS/MS data was analyzed using the Paragon algorithm in ProteinPilot 4.0 software (Applied Biosystems. USA). The raw data obtained from the machine were converted to .mgf from .wiff format file by PeakView software. To qualify and quantify the protein abundance changes of the nucleus- and organelle-encoded genes under different conditions, we searched the protein IDs and peptides mapped by ProteinPilot software against the Arabidopsis nucleus-encoded protein database, mitochondria-encoded protein database, and chloroplast-encoded protein database from the TAIR website (http://www.arabidopsis.org), respectively. Downstream analysis for the calculation of the protein expressed level for each gene from each compartment is conducted by series of in-house perl scripts. In all searches, trypsin was selected as the enzyme used for protein digestion and IAA was selected as the cysteine alkylation agent respectively. Bias correction and background correction were applied as well. For protein identifications, a minimal unused ProtScore of 1.3 with at least two peptides (confidence ≥ 95 %) was necessary. The FDR analysis was performed using the PSPEP add-on function of ProteinPilot based on a decoy database of reverse sequences. All the four replicated ratios were used for statistical analysis by using one sample *t*-test (one-tailed test). The calculate formula was listed as: $$ t={\scriptscriptstyle \frac{x-{\mu}_0}{\raisebox{1ex}{$s$}\!\left/ \!\raisebox{-1ex}{$\sqrt{n}$}\right.}} $$, where x is the mean ratio of the four replicates, μ_0_ is the assumed value (we assumed three values 1.2, 1.33 and 1.5 respectively), s is the standard deviation of the four replicates and n is the number of the replicates. The degree of freedom allowed is 3 and p < 0.05 was regarded as statistically significant.

### Quantitative RT-PCR

qRT-PCR analysis was carried out using cDNA samples transcribed from leaves harvested from 20-day-old Arabidopsis plants. Primer premier 5.0 (http://www.premierbiosoft.com/primerdesign/) was used to design the qRT-PCR primers. The PCR reactions were performed in a 10 μL volume containing a 2 × SYBR Green Master Mix (ABI systems). The amplification parameters were 95 °C for 1 min; followed by 40 cycles of 95 °C, 15 s and 60 °C 1 min. Actin 2 was used as an internal control. For every transcript, each cDNA sample was analyzed in triplicate, and relative transcript abundance was calculated by normalizing to the maximum level. The assessment of expression comparing different targets was determined by the ddCt comparative threshold (ΔΔCt) method. *P*-values were determined by two-tailed paired Student’s *t* tests.

### Validation by Western blotting

The protein samples used for proteomics were aliquoted and stored at −80 °C for western blotting. Proteins from all the three time points (T0, T1, and T8) were loaded with equal amounts and antigens were detected by specific antibodies using Enhanced Chemiluminescence method (Amersham Biosciences, UK).

### ATP/ADP/NADP^+^/NADPH extraction and measurement

Approximately 100 mg of leaves from 20-day-old Arabidopsis plants were freshly collected at three different time points (T0, T1, and T8). Adenylates were extracted by the trichloracetic acid method [47]. The ATP level was assayed using the ATP Bioluminescent Assay Kit (Sigma, FL-AA) [48]. ADP in the extract was converted into ATP by pyruvate kinase and the sum of ATP + ADP was measured [47]. The extraction of NADP^+^/NADHP was based on the selective hydrolysis of NADPH in acid medium, and selective hydrolysis of NADP^+^ in alkaline medium [49]. After pH adjustment, the levels of NADP^+^ and NADPH were measured in 96-well plates according to a plate reader based method [50]. Standard curves of 0–40 pmol pyridine nucleotides in each well were freshly prepared.

### Metabolite profiling

Metabolite profiling of Arabidopsis seedlings by GC-MS was performed as described previously [51]. Frozen leaf samples (50 mg) were homogenized in 700 μL of methanol and ribitol (0.2 mg/ml in water) was added as an internal quantitative standard for the polar phase. After centrifugation at 10,000 g for 15 min, 375 μL of chloroform and 750 μL H_2_O were added and mixed. After centrifugation at 10,000 g for 15 min, two aliquots of 150 μL upper phases (polar phase) were taken and dried by speed vac. 40 μl methoxyaminhydrochloride (20 mg/ml in pyridine) was added to the dried samples and shaken for 2 h at 37 °C. The sample was transferred into sample vial for GC-MS analysis before adding 70 μl MSTFA mix (1 ml + 20 μL FAME). After shaking for 30 min at 37 °C, the samples were analyzed by GC-MS (ChromaTOF software, Pegasus driver 1 · 61; LECO). The chromatograms and mass spectra were evaluated using TagFinder software [52]. Metabolite identification was manually supervised using the mass spectral and retention index collection of the Golm Metabolome Database [53]. Peak heights of the mass fragments were normalized on the basis of the fresh weight of the sample and the added amount of an internal standard (ribitol).

### Metabolome-based pathway activity calculation

Pathway activities for 60 pathways were calculated by the Pathway Activity Profiling (PAPi) algorithm [54], based on all measured metabolites. The pathway database used in PAPi is based on the Kyoto Encyclopedia of Genes and Genomes (KEGG) [55]. All pathways not included in the pathway database for *Arabidopsis thaliana* were removed. Global pathways, as “Metabolic pathways”, were not included in the analysis. Pathway activities were computed for each biological replicate of metabolome data, with means and SD calculated for each sample. T-test was carried out for the three pairwise comparisons (T1:T0, T8:T0 and T8:T1) to identify significantly differentially expressed pathways (*P* < 0.05).

## Availability of data and materials

The datasets supporting the conclusions of this article are included within the article and its additional files. All the raw and processed RNA-seq data were deposited in NCBI GEO (http://www.ncbi.nlm.nih.gov/geo/) with accession number (GSE57791).

## Additional files

Additional file 1:
**Total list of all transcripts in all the three samples mapped to Arabidopsis TAIR 10.0 genome.** (XLSX 4615 kb) Additional file 2:
**Transcriptional and translational profiles of chloroplast-encoded genes.** (XLSX 33 kb) Additional file 3:
**Transcriptional and translational profiles of mitochondrion-encoded genes.** (XLSX 33 kb) Additional file 4:
**Total list of significant DEGs at (a) T1 compared with T0, (b) T8 compared with T0 and (c) T8 compared with T1.** (XLSX 1863 kb) Additional file 5:
**Alternative splicing events discovered by RNA-seq.** Blue bars indicated numbers of genes with alternative splicing events and red ones indicated alternative splicing event number in each sample. (DOCX 94 kb) Additional file 6:
**Number of novel transcripts in each sample at T0, T1 and T8.** (DOCX 13 kb) Additional file 7:
**Validation of candidate genes in photosystem of wild type Arabidopsis by qRT-PCR.** All the values were calculated by fold change of the value in two compared time points. (A) indicated RNA-seq data and (B) indicated qRT-PCR data. Data were expressed as means with ± SD of three biological replicates. The significant changes in T1 and T8 were compared with T0, respectively. Asterisks indicate significant difference, **P* < 0.05, ***P* < 0.01. (DOCX 212 kb) Additional file 8:
**Peptides fractions separated profiles by SCX.** The above one shown OD220, OD260 and OD280 profiles, respectively. (DOCX 66 kb) Additional file 9:
**(A). Identification yields of protein, peptide and spectral at different FDR threshold by ProteinPilot.** (B). Correspondence between FDR levels and ID yield percentage reported by ProteinPilot (DOCX 24 kb) Additional file 10:
**Proteomics data of nuclear-encoded proteins.** (XLSX 1353 kb) Additional file 11:
**Validation of selected proteins in photosystem by western blotting.** (DOCX 153 kb) Additional file 12:
**Transcriptional and translational profiles of photosystem.** (XLSX 36 kb) Additional file 13:
**Transcriptional and translational profiles of CBB enzymes.** (XLSX 17 kb) Additional file 14:
**Transcriptional and translational profiles of carbohydrate metabolic enzymes.** (XLSX 19 kb) Additional file 15:
**Transcriptional and translational profiles of glycolysis enzymes.** (XLSX 16 kb) Additional file 16:
**Transcriptional and translational profiles of TCA cycle enzymes.** (XLSX 15 kb) Additional file 17:
**Transcriptional and translational profiles of redox proteins.** (XLSX 16 kb) Additional file 18:
**Transcriptional and translational profiles of respiratory system proteins.** (XLSX 29 kb) Additional file 19:
**Pathway activities calculated based on metabolome data.** (XLSX 24 kb) Additional file 20:
**The correlations between metabolome-based pathway activity and RNA level (left panel) or protein level (right panel).** Three pairwise comparisons based on the time points T0, T1, T8 were included. (a) The radius represents the volume (total number of genes/proteins) of the target pathway. (b) Inner circle represents the metabolome-based pathway activity; gray: no significant difference in pathway activities; red: significantly more active; green: significantly less active. (c) The intermediate ring stands for ratio of up- or down-regulated genes/proteins; grey: genes/proteins not significantly differentially expressed; red: up-regulated genes/proteins; green: down-regulated genes/proteins. (d) The outer thin ring stands for the relationship between metabolome-based pathway activity and the ratio of up- and down-regulated genes/proteins; red: positive correlation (e.g. higher metabolome- based activity and higher number of up-regulated genes), green: negative correlation (e.g. higher metabolome-based activity and higher number of down-regulated genes). (PDF 2601 kb) Additional file 21:
**Correlation between RNA and protein at a pathway level.** Three pairwise comparisons based on the T0, T1, T8 time points were included. (a) The radius represents the volume (total number of genes) of the target pathway. (b) Inner circle represents the ratio of up- and down-regulated proteins in the target pathway; gray: proteins not significantly differentially expressed; red: up-regulated proteins; green: down-regulated proteins; white: no protein detected. (c) The intermediate ring stands for ratio of up- or down-regulated genes; gray: genes not significantly differentially expressed; red: up-regulated genes; green: down-regulated genes. (d) The outer thin ring stands for the relationship between the ratios of up- and down-regulated genes and proteins in the target patwhay; red: positive correlation, green: negative correlation. (PDF 1889 kb)

## References

[1] Ma L, Li J, Qu L, Hager J, Chen Z, Zhao H (2001). Light control of Arabidopsis development entails coordinated regulation of genome expression and cellular pathways. The Plant cell.

[2] Rossel JB, Wilson IW, Pogson BJ (2002). Global changes in gene expression in response to high light in Arabidopsis. Plant Physiol.

[3] Kim BH, von Arnim AG (2006). The early dark-response in Arabidopsis thaliana revealed by cDNA microarray analysis. Plant Mol Biol.

[4] Satou M, Enoki H, Oikawa A, Ohta D, Saito K, Hachiya T (2014). Integrated analysis of transcriptome and metabolome of Arabidopsis albino or pale green mutants with disrupted nuclear-encoded chloroplast proteins. Plant Mol Biol.

[5] Covington MF, Maloof JN, Straume M, Kay SA, Harmer SL (2008). Global transcriptome analysis reveals circadian regulation of key pathways in plant growth and development. Genome Biol.

[6] Gygi SP, Rochon Y, Franza BR, Aebersold R (1999). Correlation between protein and mRNA abundance in yeast. Mol Cell Biol.

[7] Fernie AR, Stitt M (2012). On the discordance of metabolomics with proteomics and transcriptomics: coping with increasing complexity in logic, chemistry, and network interactions scientific correspondence. Plant Physiol.

[8] Motohashi R, Rodiger A, Agne B, Baerenfaller K, Baginsky S (2012). Common and specific protein accumulation patterns in different albino/pale-green mutants reveals regulon organization at the proteome level. Plant Physiol.

[9] Fiehn O, Kopka J, Dormann P, Altmann T, Trethewey RN, Willmitzer L (2000). Metabolite profiling for plant functional genomics. Nat Biotechnol.

[10] Liang C, Zhang Y, Cheng S, Osorio S, Sun Y, Fernie AR (2015). Impacts of high ATP supply from chloroplasts and mitochondria on the leaf metabolism of *Arabidopsis thaliana*. Front Plant Sci.

[11] Marquez Y, Brown JW, Simpson C, Barta A, Kalyna M (2012). Transcriptome survey reveals increased complexity of the alternative splicing landscape in Arabidopsis. Genome Res.

[12] Staiger D, Zecca L, Wieczorek Kirk DA, Apel K, Eckstein L (2003). The circadian clock regulated RNA-binding protein AtGRP7 autoregulates its expression by influencing alternative splicing of its own pre-mRNA. Plant J.

[13] Sanchez SE, Petrillo E, Beckwith EJ, Zhang X, Rugnone ML, Hernando CE (2010). A methyl transferase links the circadian clock to the regulation of alternative splicing. Nature.

[14] Palusa SG, Ali GS, Reddy ASN (2007). Alternative splicing of pre-mRNAs of Arabidopsis serine/arginine-rich proteins: regulation by hormones and stresses. Plant J.

[15] Kromer S, Heldt HW (1991). On the Role of Mitochondrial Oxidative Phosphorylation in Photosynthesis Metabolism as Studied by the Effect of Oligomycin on Photosynthesis in Protoplasts and Leaves of Barley (Hordeum vulgare). Plant Physiol.

[16] Kromer S, Malmberg G, Gardestrom P (1993). Mitochondrial Contribution to Photosynthetic Metabolism (A Study with Barley (Hordeum vulgare L.) Leaf Protoplasts at Different Light Intensities and CO2 Concentrations). Plant Physiol.

[17] Gardestrom P, Lernmark U (1995). The contribution of mitochondria to energetic metabolism in photosynthetic cells. J Bioenerg Biomembr.

[18] Igamberdiev AU, Shen T, Gardestrom P (2006). Function of mitochondria during the transition of barley protoplasts from low light to high light. Planta.

[19] Law YS, Zhang R, Guan X, Cheng S, Sun F, Duncan O (2015). Phosphorylation and Dephosphorylation of the Presequence of pMORF3 During Import into Mitochondria from Arabidopsis thaliana. Plant Physiol.

[20] Herranen M, Tyystjarvi T, Aro EM (2005). Regulation of photosystem I reaction center genes in Synechocystis sp. strain PCC 6803 during Light acclimation. Plant Cell Physiol.

[21] Adachi Y, Kuroda H, Yukawa Y, Sugiura M (2012). Translation of partially overlapping psbD-psbC mRNAs in chloroplasts: the role of 5’-processing and translational coupling. Nucleic Acids Res.

[22] Reiland S, Grossmann J, Baerenfaller K, Gehrig P, Nunes-Nesi A, Fernie AR (2011). Integrated proteome and metabolite analysis of the de-etiolation process in plastids from rice (Oryza sativa L.). Proteomics.

[23] Schuhmann H, Adamska I (2012). Deg proteases and their role in protein quality control and processing in different subcellular compartments of the plant cell. Physiol Plant.

[24] Zienkiewicz M, Ferenc A, Wasilewska W, Romanowska E (2012). High light stimulates Deg1-dependent cleavage of the minor LHCII antenna proteins CP26 and CP29 and the PsbS protein in Arabidopsis thaliana. Planta.

[25] Yagi Y, Shiina T (2014). Recent advances in the study of chloroplast gene expression and its evolution. Front Plant Sci.

[26] Baba K, Schmidt J, Espinosa-Ruiz A, Villarejo A, Shiina T, Gardestrom P (2004). Organellar gene transcription and early seedling development are affected in the rpoT;2 mutant of Arabidopsis. Plant J.

[27] Allison LA, Simon LD, Maliga P (1996). Deletion of rpoB reveals a second distinct transcription system in plastids of higher plants. EMBO J.

[28] Liere K, Weihe A, Borner T (2011). The transcription machineries of plant mitochondria and chloroplasts: Composition, function, and regulation. J Plant Physiol.

[29] Kuhn K, Richter U, Meyer EH, Delannoy E, de Longevialle AF, O’Toole N (2009). Phage-type RNA polymerase RPOTmp performs gene-specific transcription in mitochondria of Arabidopsis thaliana. Plant Cell.

[30] Kwasniak M, Majewski P, Skibior R, Adamowicz A, Czarna M, Sliwinska E (2013). Silencing of the nuclear RPS10 gene encoding mitochondrial ribosomal protein alters translation in arabidopsis mitochondria. Plant Cell.

[31] Livingston AK, Cruz JA, Kohzuma K, Dhingra A, Kramer DM (2010). An Arabidopsis mutant with high cyclic electron flow around photosystem I (hcef) involving the NADPH dehydrogenase complex. Plant Cell.

[32] Sweetlove LJ, Beard KF, Nunes-Nesi A, Fernie AR, Ratcliffe RG (2010). Not just a circle: flux modes in the plant TCA cycle. Trends Plant Sci.

[33] Cheung CY, Poolman MG, Fell DA, Ratcliffe RG, Sweetlove LJ (2014). A Diel Flux Balance Model Captures Interactions between Light and Dark Metabolism during Day-Night Cycles in C3 and Crassulacean Acid Metabolism Leaves. Plant Physiol.

[34] Poolman MG, Miguet L, Sweetlove LJ, Fell DA (2009). A genome-scale metabolic model of Arabidopsis and some of its properties. Plant Physiol.

[35] Szecowka M, Heise R, Tohge T, Nunes-Nesi A, Vosloh D, Huege J (2013). Metabolic fluxes in an illuminated Arabidopsis rosette. Plant Cell.

[36] Nunes-Nesi A, Araujo WL, Obata T, Fernie AR (2013). Regulation of the mitochondrial tricarboxylic acid cycle. Curr Opin Plant Biol.

[37] Barakat A, Szick-Miranda K, Chang IF, Guyot R, Blanc G, Cooke R (2001). The organization of cytoplasmic ribosomal protein genes in the Arabidopsis genome. Plant Physiol.

[38] Liu MJ, Wu SH, Chen HM (2012). Widespread translational control contributes to the regulation of Arabidopsis photomorphogenesis. Mol Syst Biol.

[39] Narsai R, Howell KA, Millar AH, O’Toole N, Small I, Whelan J (2007). Genome-wide analysis of mRNA decay rates and their determinants in Arabidopsis thaliana. Plant Cell.

[40] Valencia-Sanchez MA, Liu J, Hannon GJ, Parker R (2006). Control of translation and mRNA degradation by miRNAs and siRNAs. Gene Dev.

[41] Li R, Yu C, Li Y, Lam TW, Yiu SM, Kristiansen K (2009). SOAP2: an improved ultrafast tool for short read alignment. Bioinformatics.

[42] Mortazavi A, Williams BA, McCue K, Schaeffer L, Wold B (2008). Mapping and quantifying mammalian transcriptomes by RNA-Seq. Nat Methods.

[43] Huang S, Zhang J, Li R, Zhang W, He Z, Lam TW (2011). SOAPsplice: Genome-Wide ab initio Detection of Splice Junctions from RNA-Seq Data. Front Genet.

[44] Bradford MM (1976). A rapid and sensitive method for the quantitation of microgram quantities of protein utilizing the principle of protein-dye binding. Anal Biochem.

[45] Ross PL, Huang YN, Marchese JN, Williamson B, Parker K, Hattan S (2004). Multiplexed protein quantitation in Saccharomyces cerevisiae using amine-reactive isobaric tagging reagents. Mol Cell Proteomics.

[46] Zhao Y, Kong RP, Li G, Lam MP, Law CH, Lee SM (2012). Fully automatable two-dimensional hydrophilic interaction liquid chromatography-reversed phase liquid chromatography with online tandem mass spectrometry for shotgun proteomics. J Sep Sci.

[47] Meyer EH, Tomaz T, Carroll AJ, Estavillo G, Delannoy E, Tanz SK (2009). Remodeled respiration in ndufs4 with low phosphorylation efficiency suppresses Arabidopsis germination and growth and alters control of metabolism at night. Plant Physiol.

[48] Ford SR, Leach FR (1998). Bioluminescent assay of the adenylate energy charge. Methods Mol Biol.

[49] Foyer C, Lelandais M, Galap C, Kunert KJ (1991). Effects of Elevated Cytosolic Glutathione Reductase Activity on the Cellular Glutathione Pool and Photosynthesis in Leaves under Normal and Stress Conditions. Plant Physiol.

[50] Queval G, Noctor G (2007). A plate reader method for the measurement of NAD, NADP, glutathione, and ascorbate in tissue extracts: Application to redox profiling during Arabidopsis rosette development. Anal Biochem.

[51] Lisec J, Schauer N, Kopka J, Willmitzer L, Fernie AR (2006). Gas chromatography mass spectrometry-based metabolite profiling in plants. Nat Protoc.

[52] Luedemann A, von Malotky L, Erban A, Kopka J (2012). TagFinder: preprocessing software for the fingerprinting and the profiling of gas chromatography-mass spectrometry based metabolome analyses. Methods Mol Biol.

[53] Kopka J, Schauer N, Krueger S, Birkemeyer C, Usadel B, Bergmuller E (2005). GMD@CSB.DB: the Golm Metabolome Database. Bioinformatics.

[54] Aggio RB, Ruggiero K, Villas-Boas SG (2010). Pathway Activity Profiling (PAPi): from the metabolite profile to the metabolic pathway activity. Bioinformatics.

[55] Kanehisa M, Goto S (2000). KEGG: kyoto encyclopedia of genes and genomes. Nucleic Acids Res.

