# Supplementary material for: Transcriptomic, proteomic and metabolic changes in Arabidopsis thaliana leaves after the onset of illumination
Source: BMC Plant Biol. 2016 Feb 11;16:43. doi: 10.1186/s12870-016-0726-3 (PMC4750186; doi:10.1186/s12870-016-0726-3)
Supplement: Additional file 5: — Alternative splicing events discovered by RNA-seq. Blue bars indicated numbers of genes with alternative splicing events and red ones indicated alternative splicing event number in each sample. (DOCX 94 kb) [file 12870_2016_726_MOESM5_ESM.docx]

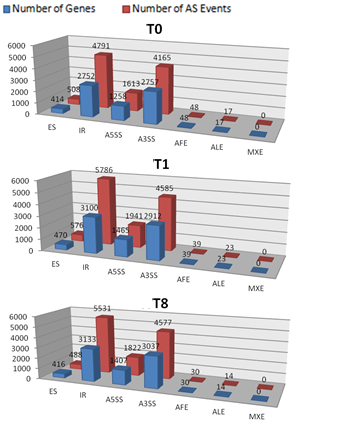


**Additional file 5. Alternative splicing events discovered by RNA-seq.** Blue bars indicated numbers of genes with alternative splicing events and red ones indicated alternative splicing event number in each sample.
