# Supplementary material for: Transcriptomic, proteomic and metabolic changes in Arabidopsis thaliana leaves after the onset of illumination
Source: BMC Plant Biol. 2016 Feb 11;16:43. doi: 10.1186/s12870-016-0726-3 (PMC4750186; doi:10.1186/s12870-016-0726-3)
Supplement: Additional file 6: — Number of novel transcripts in each sample at T0, T1 and T8. (DOCX 13 kb) [file 12870_2016_726_MOESM6_ESM.docx]

**Additional file 6. Number of novel transcripts in each sample at T0, T1 and T8.**
