# Supplementary material for: Transcriptomic, proteomic and metabolic changes in Arabidopsis thaliana leaves after the onset of illumination
Source: BMC Plant Biol. 2016 Feb 11;16:43. doi: 10.1186/s12870-016-0726-3 (PMC4750186; doi:10.1186/s12870-016-0726-3)
Supplement: Additional file 7: — Validation of candidate genes in photosystem of wild type Arabidopsis by qRT-PCR. All the values were calculated by fold change of the value in two compared time points. (A) indicated RNA-seq data and (B) indicated qRT-PCR data. Data were expressed as means with ± SD of three biological replicates. The significant changes in T1 and T8 were compared with T0, respectively. Asterisks indicate significant difference, *P < 0.05, **P < 0.01. (DOCX 212 kb) [file 12870_2016_726_MOESM7_ESM.docx]

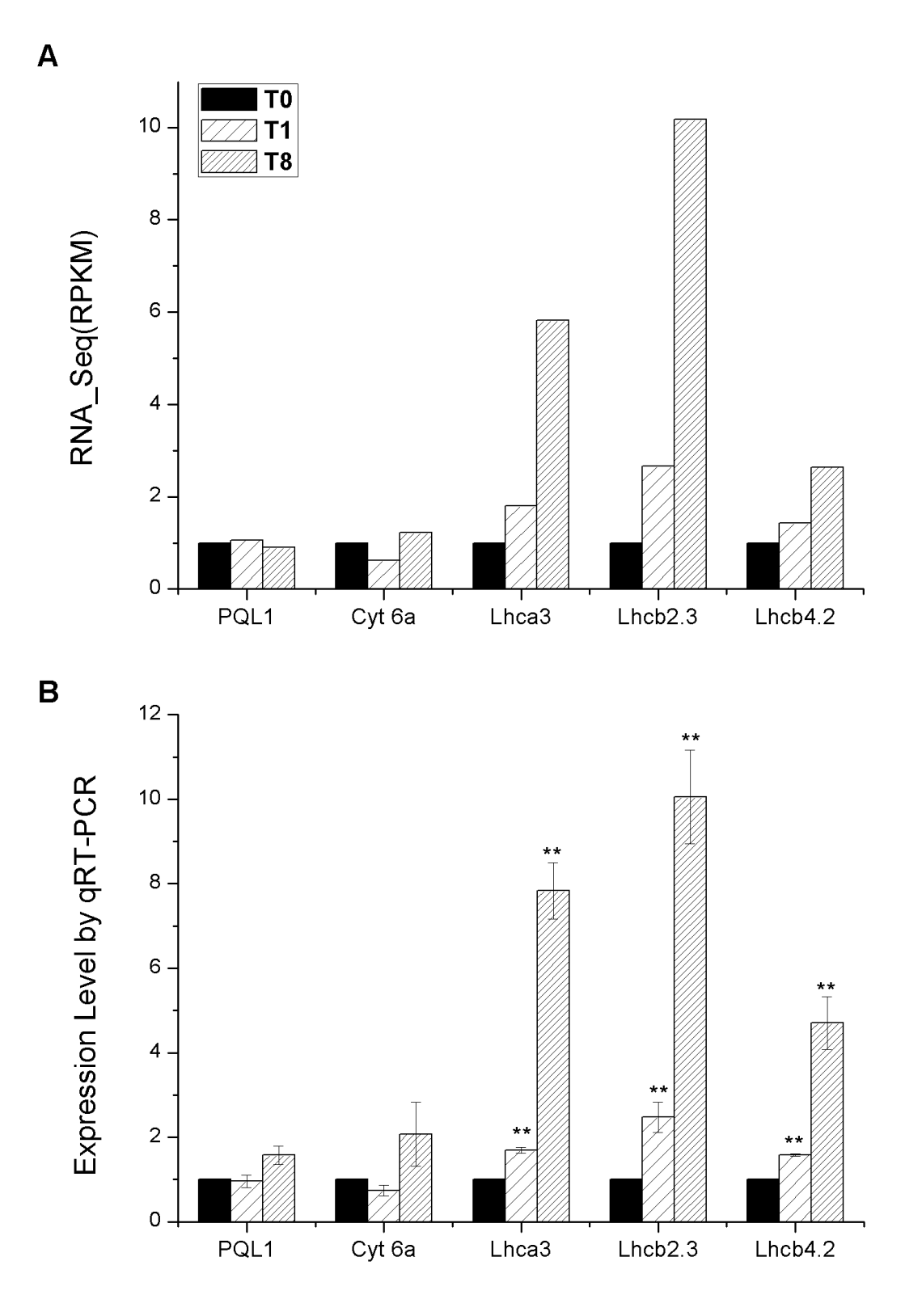


**Additional file 7. Validation of candidate genes in photosystem of wild type Arabidopsis by qRT-PCR. All the values were calculated by fold change of the value in two compared time points. (A)** indicated RNA-seq data and **(B)** indicated qRT-PCR data. Data were expressed as means with ±SD of three biological replicates. The significant changes in T1 and T8 were compared with T0, respectively. Asterisks indicate significant difference, *P<0.05, **P<0.01.
