# Supplementary material for: Transcriptomic, proteomic and metabolic changes in Arabidopsis thaliana leaves after the onset of illumination
Source: BMC Plant Biol. 2016 Feb 11;16:43. doi: 10.1186/s12870-016-0726-3 (PMC4750186; doi:10.1186/s12870-016-0726-3)
Supplement: Additional file 8: — Peptides fractions separated profiles by SCX. The above one shown OD220, OD260 and OD280 profiles, respectively. (DOCX 66 kb) [file 12870_2016_726_MOESM8_ESM.docx]

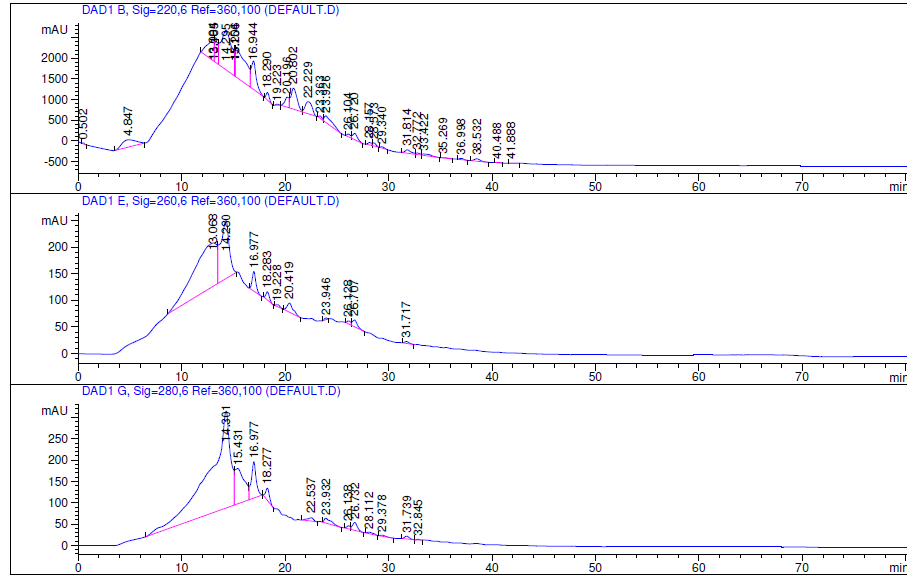


**Additional file 8. Peptides fractions separated profiles by SCX.** The above one shown OD220, OD260 and OD280 profiles, respectively.
