# Supplementary material for: Transcriptomic, proteomic and metabolic changes in Arabidopsis thaliana leaves after the onset of illumination
Source: BMC Plant Biol. 2016 Feb 11;16:43. doi: 10.1186/s12870-016-0726-3 (PMC4750186; doi:10.1186/s12870-016-0726-3)
Supplement: Additional file 9: — (A). Identification yields of protein, peptide and spectral at different FDR threshold by ProteinPilot. (B). Correspondence between FDR levels and ID yield percentage reported by ProteinPilot (DOCX 24 kb) [file 12870_2016_726_MOESM9_ESM.docx]

**Additional file 9 (A). Identification yields of protein, peptide and spectral at different FDR threshold by ProteinPilot**

| **Data Level** | **FDR Type** | **FDR** | **ID Yield** |
| --- | --- | --- | --- |
| **Protein** | Local | 1% | 2,346 |
|  |  | **5%** | **2,689** |
|  |  | 10% | 2,803 |
|  | Global | **1%** | **2,872** |
|  |  | 5% | 3,229 |
|  |  | 10% | 3,444 |
| **Distinct peptide** | Local | 1% | 14,737 |
|  |  | **5%** | **19,381** |
|  |  | 10% | 21,601 |
|  | Global | **1%** | **20,343** |
|  |  | 5% | 26,531 |
|  |  | 10% | 30,621 |
| **Spectral** | Local | 1% | 72,101 |
|  |  | **5%** | **81,481** |
|  |  | 10% | 92,765 |
|  | Global | **1%** | **91,147** |
|  |  | 5% | 128,640 |
|  |  | 10% | - |

Note: Local FDR indicated the FDR of an individual protein, peptide or spectral and Global FDR represented the FDR applied to the entire set of proteins, peptides or spectrals. FDR and ID yield in bold were the criteria for data analysis.

**Additional file 9 (B). Correspondence between FDR levels and ID yield percentage reported by ProteinPilot**

| **Data Level** | **FDR Type** | **FDR** | **ID yield percentage** |
| --- | --- | --- | --- |
| **Protein** | Local | 1% | 99.0% |
|  |  | **5%** | **97.0%** |
|  |  | 10% | 93.2% |
|  | Global | **1%** | **88.8%** |
|  |  | 5% | 58.3% |
|  |  | 10% | 42.5% |
| **Distinct peptide** | Local | 1% | 99.4% |
|  |  | **5%** | **95.1%** |
|  |  | 10% | 86.0% |
|  | Global | **1%** | **92.4%** |
|  |  | 5% | 45.7% |
|  |  | 10% | 20.6% |
| **Spectral** | Local | 1% | 99.2% |
|  |  | **5%** | **97.8%** |
|  |  | 10% | 93.5% |
|  | Global | **1%** | **94.4%** |
|  |  | 5% | 36.6% |
|  |  | 10% | - |

Note: FDR and ID yield in bold were the criteria for data analysis.
