# Supplementary material for: Transcriptomic, proteomic and metabolic changes in Arabidopsis thaliana leaves after the onset of illumination
Source: BMC Plant Biol. 2016 Feb 11;16:43. doi: 10.1186/s12870-016-0726-3 (PMC4750186; doi:10.1186/s12870-016-0726-3)
Supplement: Additional file 11: — Validation of selected proteins in photosystem by western blotting. (DOCX 153 kb) [file 12870_2016_726_MOESM11_ESM.docx]

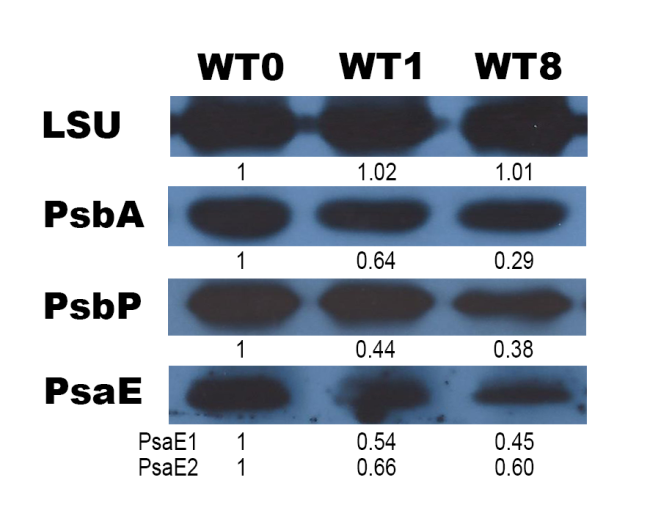


**Additional file 11. Validation of selected proteins in photosystem by western blotting.** To validate the proteomics data, the abundance of selected proteins in photosystem (PsaE, PsbA, PsbP) and LSU (large subunit of RuBisCO) were compared among WT0, WT1, WT8 by western blotting. WT0 was set as a control for comparison and LSU protein was set as the housekeeping protein. The values were ratios that were presented in proteomics data (**Additional_file_12**). Western blot results showed a close correlation with proteomics data.
