# Supplementary material for: Transcriptomic, proteomic and metabolic changes in Arabidopsis thaliana leaves after the onset of illumination
Source: BMC Plant Biol. 2016 Feb 11;16:43. doi: 10.1186/s12870-016-0726-3 (PMC4750186; doi:10.1186/s12870-016-0726-3)
Supplement: Additional file 20: — The correlations between metabolome-based pathway activity and RNA level (left panel) or protein level (right panel). Three pairwise comparisons based on the time points T0, T1, T8 were included. (a) The radius represents the volume (total number of genes/proteins) of the target pathway. (b) Inner circle represents the metabolome-based pathway activity; gray: no significant difference in pathway activities; red: significantly more active; green: significantly less active. (c) The intermediate ring stands for ratio of up- or down-regulated genes/proteins; grey: genes/proteins not significantly differentially expressed; red: up-regulated genes/proteins; green: down-regulated genes/proteins. (d) The outer thin ring stands for the relationship between metabolome-based pathway activity and the ratio of up- and down-regulated genes/proteins; red: positive correlation (e.g. higher metabolome- based activity and higher number of up-regulated genes), green: negative correlation (e.g. higher metabolome-based activity and higher number of down-regulated genes). (PDF 2601 kb) [file 12870_2016_726_MOESM20_ESM.pdf]

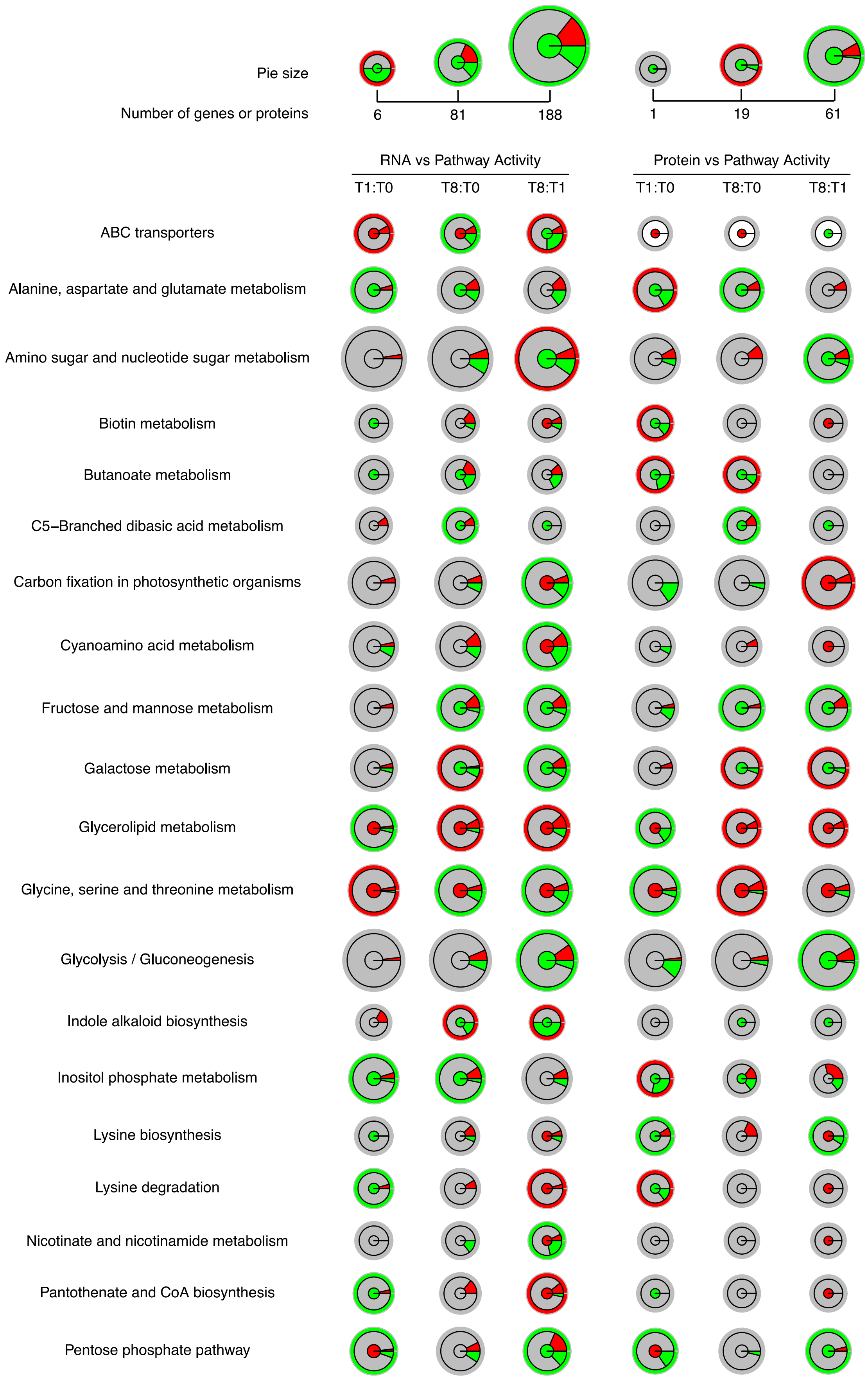

Pie size
Number of genes or proteins
6
81
188
1
19
61
RNA vs Pathway Activity
Protein vs Pathway Activity
T1:T0
T8:T0
T8:T1
T1:T0
T8:T0
T8:T1
ABC transporters
Alanine, aspartate and glutamate metabolism
Amino sugar and nucleotide sugar metabolism
Biotin metabolism
Butanoate metabolism
C5−Branched dibasic acid metabolism
Carbon fixation in photosynthetic organisms
Cyanoamino acid metabolism
Fructose and mannose metabolism
Galactose metabolism
Glycerolipid metabolism
Glycine, serine and threonine metabolism
Glycolysis / Gluconeogenesis
Indole alkaloid biosynthesis
Inositol phosphate metabolism
Lysine biosynthesis
Lysine degradation
Nicotinate and nicotinamide metabolism
Pantothenate and CoA biosynthesis
Pentose phosphate pathway

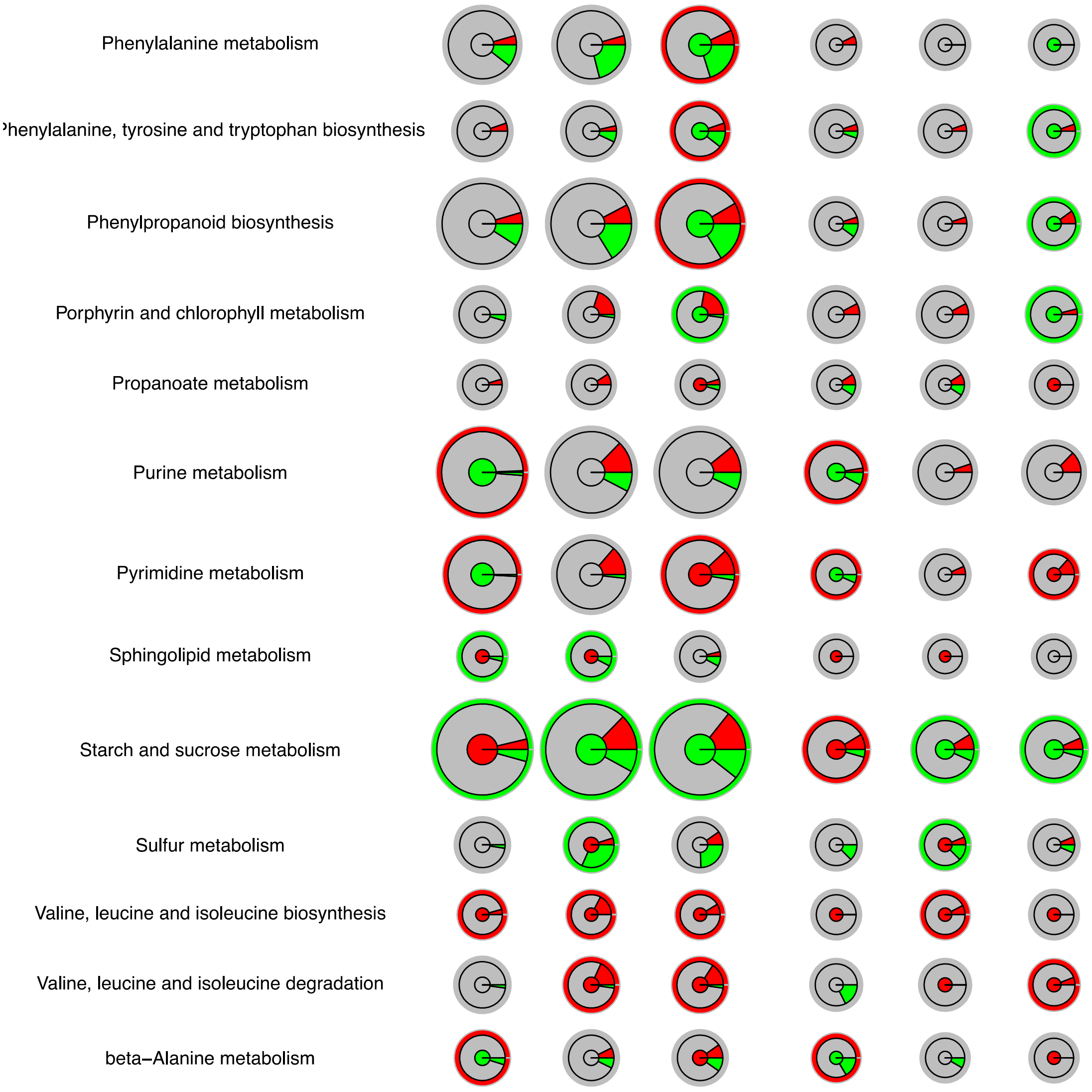

Additional file 20. The correlations between metabolome-based pathway activity and RNA level (left panel) or protein level (right panel). Three pairwise comparisons based on the time points T0, T1, T8 were included. (a) The radius represents the volume (total number of genes/proteins) of the target pathway. (b) Inner circle represents the metabolome-based pathway activity; gray: no significant difference in pathway activities; red: significantly more active; green: significantly less active. (c) The intermediate ring stands for ratio of up- or down-regulated genes/proteins; grey: genes/proteins not significantly differentially expressed; red: up-regulated genes/proteins; green: down-regulated genes/proteins. (d) The outer thin ring stands for the relationship between metabolome-based pathway activity and the ratio of up- and down-regulated genes/proteins; red: positive correlation (e.g. higher

metabolome-based activity and higher number of up-regulated genes), green: negative correlation (e.g. higher metabolome-based activity and higher number of down-regulated genes).
