# Supplementary material for: Transcriptomic, proteomic and metabolic changes in Arabidopsis thaliana leaves after the onset of illumination
Source: BMC Plant Biol. 2016 Feb 11;16:43. doi: 10.1186/s12870-016-0726-3 (PMC4750186; doi:10.1186/s12870-016-0726-3)
Supplement: Additional file 21: — Correlation between RNA and protein at a pathway level. Three pairwise comparisons based on the T0, T1, T8 time points were included. (a) The radius represents the volume (total number of genes) of the target pathway. (b) Inner circle represents the ratio of up- and down-regulated proteins in the target pathway; gray: proteins not significantly differentially expressed; red: up-regulated proteins; green: down-regulated proteins; white: no protein detected. (c) The intermediate ring stands for ratio of up- or down-regulated genes; gray: genes not significantly differentially expressed; red: up-regulated genes; green: down-regulated genes. (d) The outer thin ring stands for the relationship between the ratios of up- and down-regulated genes and proteins in the target patwhay; red: positive correlation, green: negative correlation. (PDF 1889 kb) [file 12870_2016_726_MOESM21_ESM.pdf]

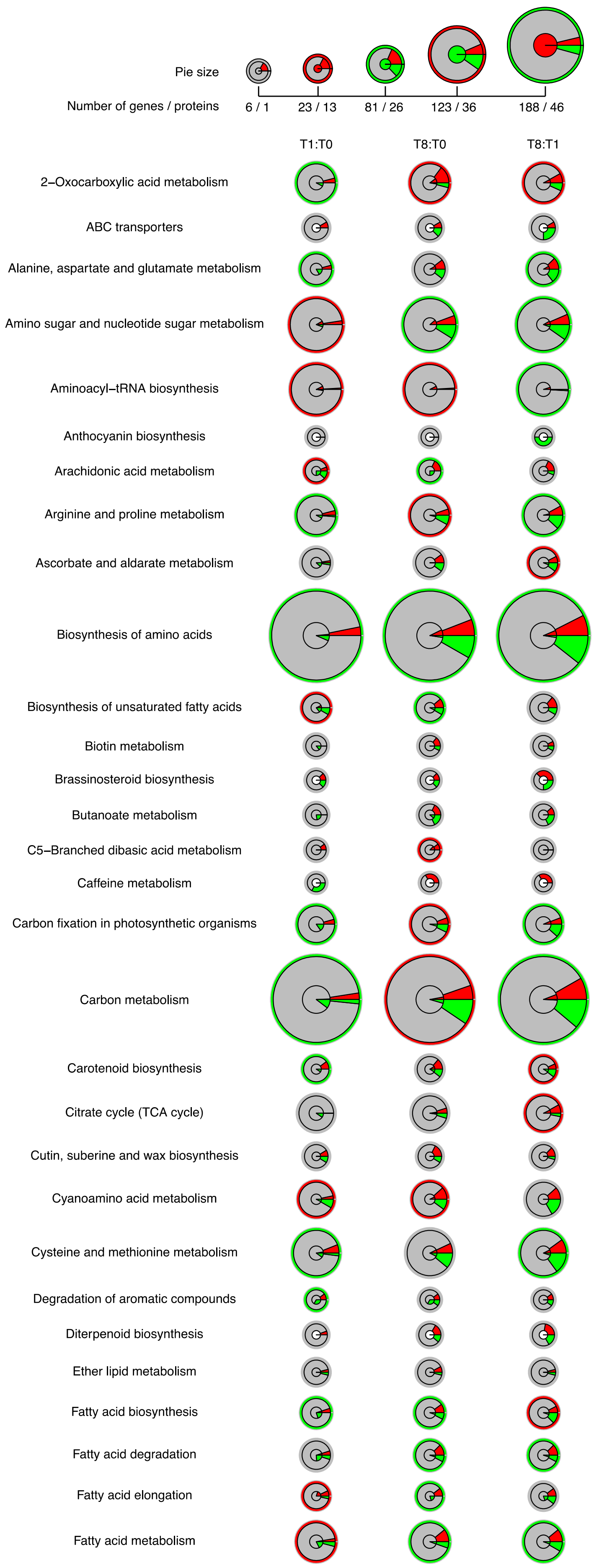

Pie size
Number of genes / proteins
6 / 1
23 / 13
81 / 26
123 / 36
188 / 46
T1:T0
T8:T0
T8:T1
2−Oxocarboxylic acid metabolism
ABC transporters
Alanine, aspartate and glutamate metabolism
Amino sugar and nucleotide sugar metabolism
Aminoacyl−tRNA biosynthesis
Anthocyanin biosynthesis
Arachidonic acid metabolism
Arginine and proline metabolism
Ascorbate and aldarate metabolism
Biosynthesis of amino acids
Biosynthesis of unsaturated fatty acids
Biotin metabolism
Brassinosteroid biosynthesis
Butanoate metabolism
C5−Branched dibasic acid metabolism
Caffeine metabolism
Carbon fixation in photosynthetic organisms
Carbon metabolism
Carotenoid biosynthesis
Citrate cycle (TCA cycle)
Cutin, suberine and wax biosynthesis
Cyanoamino acid metabolism
Cysteine and methionine metabolism
Degradation of aromatic compounds
Diterpenoid biosynthesis
Ether lipid metabolism
Fatty acid biosynthesis
Fatty acid degradation
Fatty acid elongation
Fatty acid metabolism

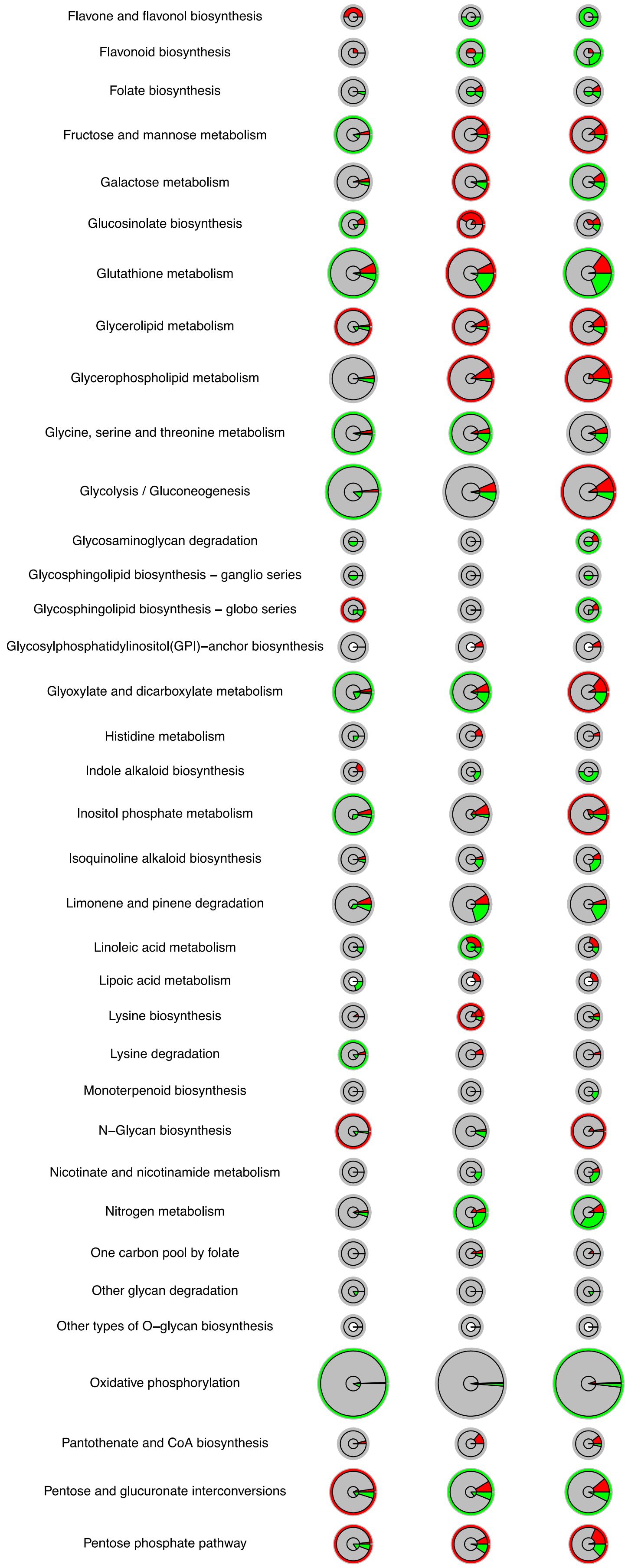
Flavone and flavonol biosynthesis
Flavonoid biosynthesis
Folate biosynthesis
Fructose and mannose metabolism
Galactose metabolism
Glucosinolate biosynthesis
Glutathione metabolism
Glycerolipid metabolism
Glycerophospholipid metabolism
Glycine, serine and threonine metabolism
Glycolysis / Gluconeogenesis
Glycosaminoglycan degradation
Glycosphingolipid biosynthesis – ganglio series
Glycosphingolipid biosynthesis – globo series
Glycosylphosphatidylinositol(GPI)–anchor biosynthesis
Glyoxylate and dicarboxylate metabolism
Histidine metabolism
Indole alkaloid biosynthesis
Inositol phosphate metabolism
Isoquinoline alkaloid biosynthesis
Limonene and pinene degradation
Linoleic acid metabolism
Lipoic acid metabolism
Lysine biosynthesis
Lysine degradation
Monoterpenoid biosynthesis
N–Glycan biosynthesis
Nicotinate and nicotinamide metabolism
Nitrogen metabolism
One carbon pool by folate
Other glycan degradation
Other types of O–glycan biosynthesis
Oxidative phosphorylation
Pantothenate and CoA biosynthesis
Pentose and glucuronate interconversions
Pentose phosphate pathway

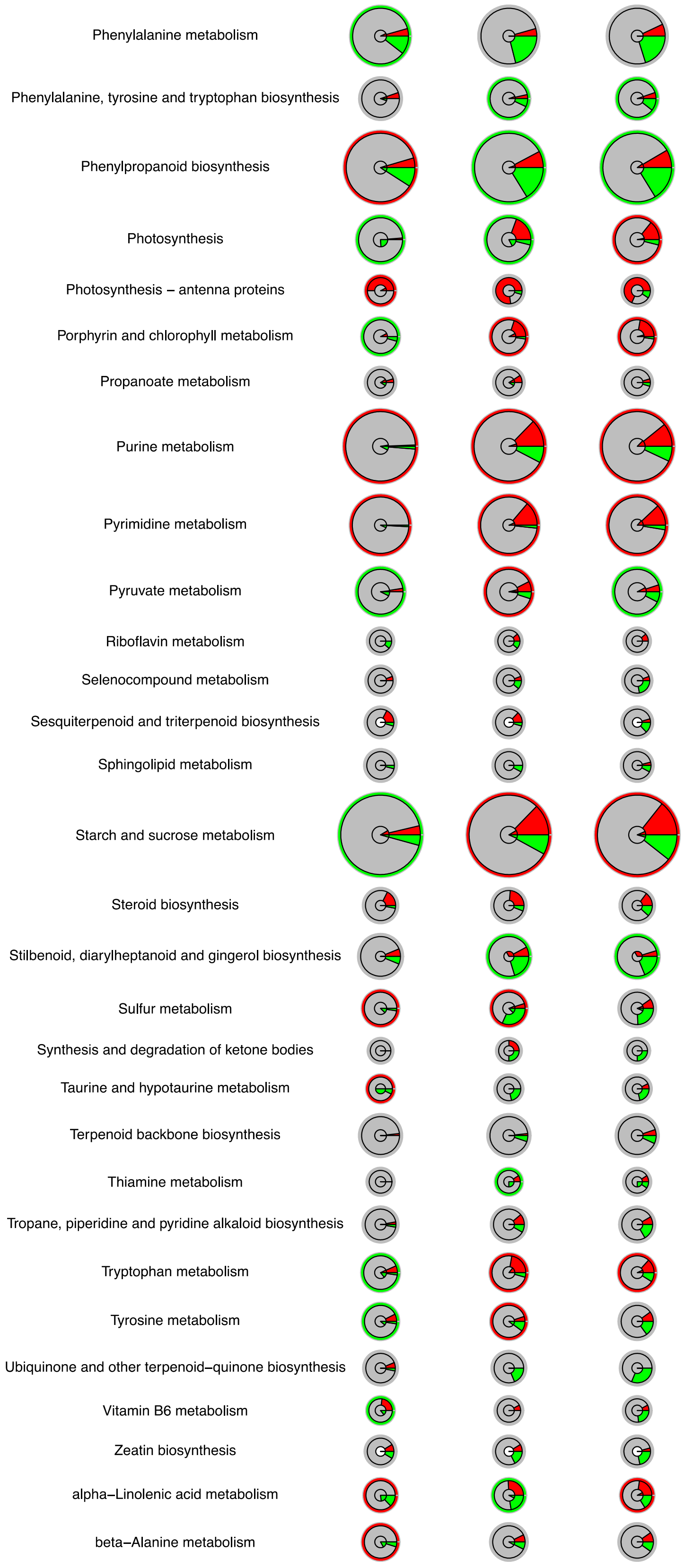

Additional file 21. Correlation between RNA and protein at a pathway level. Three pairwise comparisons based on the T0, T1, T8 time points were included. (a) The radius represents the volume (total number of genes) of the target pathway. (b)

Inner circle represents the ratio of up- and down-regulated proteins in the target pathway; gray: proteins not significantly differentially expressed; red: up-regulated proteins; green: down-regulated proteins; white: no protein detected. (c) The intermediate ring stands for ratio of up- or down-regulated genes; gray: genes not significantly differentially expressed; red: up-regulated genes; green: down-regulated genes. (d) The outer thin ring stands for the relationship between the ratios of up- and down-regulated genes and proteins in the target patwhay; red: positive correlation, green: negative correlation.
